# The long non-coding RNA *LINC00707* interacts with Smad proteins to regulate TGFβ signaling and cancer cell invasion

**DOI:** 10.1186/s12964-023-01273-3

**Published:** 2023-10-02

**Authors:** Caroline Gélabert, Panagiotis Papoutsoglou, Irene Golán, Eric Ahlström, Adam Ameur, Carl-Henrik Heldin, Laia Caja, Aristidis Moustakas

**Affiliations:** 1 Department of Medical Biochemistry and Microbiology, Science for Life Laboratory, Biomedical Center, Uppsala University, Box 582, Uppsala, SE-75123 Sweden; 2 Inserm, Centre de Lutte contre le Cancer Eugène Marquis, Université Rennes 1, OSS (Oncogenesis, Stress, Signalling) laboratory, UMR_S 1242, Rennes, F-35042 France; 3 Science for Life Laboratory, Department of Immunology, Genetics and Pathology, Uppsala University, Uppsala, Sweden

**Keywords:** Cell invasion, lncRNA, Signal transduction, RNA-protein interaction, Transforming growth factor (TGFβ)

## Abstract

**Background:**

Long non-coding RNAs (lncRNAs) regulate cellular processes by interacting with RNAs or proteins. Transforming growth factor β (TGFβ) signaling via Smad proteins regulates gene networks that control diverse biological processes, including cancer cell migration. LncRNAs have emerged as TGFβ targets, yet, their mechanism of action and biological role in cancer remain poorly understood.

**Methods:**

Whole-genome transcriptomics identified lncRNA genes regulated by TGFβ. Protein kinase inhibitors and RNA-silencing, in combination with cDNA cloning, provided loss- and gain-of-function analyses. Cancer cell-based assays coupled to RNA-immunoprecipitation, chromatin isolation by RNA purification and protein screening sought mechanistic evidence. Functional validation of TGFβ-regulated lncRNAs was based on new transcriptomics and by combining RNAscope with immunohistochemical analysis in tumor tissue.

**Results:**

Transcriptomics of TGFβ signaling responses revealed down-regulation of the predominantly cytoplasmic *long intergenic non-protein coding RNA 707* (*LINC00707*). Expression of *LINC00707* required Smad and mitogen-activated protein kinase inputs. By limiting the binding of Krüppel-like factor 6 to the *LINC00707* promoter, TGFβ led to *LINC00707* repression. Functionally, *LINC00707* suppressed cancer cell invasion, as well as key fibrogenic and pro-mesenchymal responses to TGFβ, as also attested by RNA-sequencing analysis. *LINC00707* also suppressed Smad-dependent signaling. Mechanistically, *LINC00707* interacted with and retained Smad proteins in the cytoplasm. Upon TGFβ stimulation, *LINC00707* dissociated from the Smad complex, which allowed Smad accumulation in the nucleus. In vivo, *LINC00707* expression was negatively correlated with Smad2 activation in tumor tissues.

**Conclusions:**

*LINC00707* interacts with Smad proteins and limits the output of TGFβ signaling, which decreases *LINC00707* expression, thus favoring cancer cell invasion.

Video Abstract

**Supplementary Information:**

The online version contains supplementary material available at 10.1186/s12964-023-01273-3.

## Background

The TGFβ family of ligands regulates several biological processes, such as embryonic development, adult tissue homeostasis, differentiation and migration [1, 2]. In cancer, TGFβ suppresses tumorigenic evolution of premalignant lesions by promoting apoptosis; however, in advanced malignancies, TGFβ promotes tumor cell migration and metastasis [1, 3].

Signaling initiates by binding of TGFβ ligands to specific receptors (type I, TβRI and type II, TβRII) which possess serine, threonine and tyrosine kinase activity [4]. Ligand-bound TβRII recruits, phosphorylates and activates TβRI, allowing TβRI to phosphorylate effector Smad family transcription factors [2, 3]. The TGFβ Smad family includes two receptor-activated Smads (R-Smads, Smad2 and Smad3), one common mediator (Co-Smad, Smad4) and two inhibitory Smads (I-Smads, Smad6 and Smad7). Phosphorylated R-Smads accumulate in the nucleus as they make complexes with Smad4. In contrast, the I-Smads quench the activation of the pathway, thus fine-tuning TGFβ signaling [2, 5]. In addition to Smads, the TGFβ receptor complex activates alternative pathways, such as the mitogen-activated protein kinases (MAPKs), the tyrosine kinase Src and the lipid kinase phosphatidylinositol-3´kinase, that mediate cytoplasmic signaling and control Smad activity [2]. The nuclear Smad complex and the associated non-Smad kinases affect either positively or negatively the expression of many protein-coding and non-coding genes in response to TGFβ. Among the best characterized TGFβ-target protein-coding genes are *fibronectin-1* (*FN1*) [6] and *serpine-1* (*SERPINE1*) [7]. The TGFβ pathway affects expression of almost as many lncRNAs as mRNAs [8–10], including the *lncRNA-activated by TGFβ* (*lncRNA-ATB*) [11] and the lncRNA *HOXA antisense transcript induced by TGFβ* (*lncRNA-HIT*) [12]. Furthermore, lncRNAs whose expression is regulated by TGFβ can regulate the TGFβ pathway [9, 10], with a recent example the chromatin-associated *TGFβ2-AS1* lncRNA [13]. Such lncRNAs are frequently overexpressed in diverse human tumors, as for example the *lncRNA-MUF/LINC00941* and *LINC01711* that are overexpressed in glioblastoma tumors, both being highly regulated by TGFβ signaling, and acting as sponges/competing endogenous RNAs to the miRNA *miR-34a*, a negative regulator of transcription factors SNAI1 and ZEB1, thus promoting tumor cell invasiveness [14, 15].

LncRNAs represent a class of non-coding RNAs, longer than 200 nucleotides, lacking functional open reading frames. LncRNAs and mRNAs share similarities in their synthesis by RNA polymerase II, their splicing and post-transcriptional modifications. Depending on their genomic localization, relative to other genes, lncRNAs are classified as natural antisense, bidirectional, intronic or intergenic transcripts [16]. Functionally, they regulate gene expression at a transcriptional, post-transcriptional or post-translational level. In addition, they participate in epigenetic modification of genes, by interacting with chromatin-modifying enzymes [17].

Here, we show that TGFβ signaling represses *LINC00707* expression via specific transcriptional mechanisms, and *LINC00707* forms complexes with Smads and limits TGFβ signaling based on cytoplasmic retention of Smads. Thus, long-term suppression of *LINC00707* expression by TGFβ enhances pro-tumorigenic TGFβ signaling.

## Methods

### Cell culture

Human immortalized keratinocytes (HaCaT), human lung adenocarcinoma cells (A549 and H1299) and breast cancer cells (MDA-MB-231, MCF-10 A) were cultured in Dulbecco´s modified Eagle´s medium (DMEM; Sigma-Aldrich AB, Stockholm, Sweden) supplemented with 10% fetal bovine serum (FBS; Biowest, Almeco A/S, Esbjerg, Denmark). Human prostate cancer PC-3U cells (originating from the PC-3 cell line) were grown in RPMI-1640 medium with GlutaMax^TM^-l (Gibco, Life Technologies Ltd, Paisley, UK), and 10% FBS (Gibco, Life Technologies Ltd, Paisley, UK). The human glioblastoma cell line U2987MG was cultured in MEM with 10% FBS, and the human glioblastoma cell lines U3031MG and U3034MG were acquired from the Human Glioblastoma cell Culture resource (www.hgcc.se) at the Dept. of Immunology, Genetics and Pathology, Uppsala University, Sweden [18]. They were cultured in N2B27 media, made of DMEM/F12 with Glutamax (ThermoFischer Scientific, Uppsala, Sweden) and neurobasal medium mixed at 1:1 ratio, with the addition of 1% B27 and 1% N2 (ThermoFischer Scientific, Uppsala, Sweden), 100 U/ml penicillin and 100 mg/ml streptomycin (Sigma-Aldrich Sweden AB, Stockholm, Sweden), 10 ng/ml EGF and 10 ng/ml FGF2 (PeproTech, EC Ltd, London, UK). Glioblastoma cells were seeded onto poly-ornithine/laminin-coated dishes to be cultured as adherent cells. All cell lines were kept in humidified incubators at 37 °C and 5% CO_2_.

### Treatments and inhibitors

Cells were starved overnight in serum-free RPMI or DMEM medium before treatment with recombinant human TGFβ1 (5 ng/ml, PeproTech EC Ltd, London, UK), recombinant human BMP4 (30 ng/mL, PeproTech EC Ltd, London, UK), recombinant human BMP7 (30 ng/mL, a gift from Kuber Sampath, Sanofi-Genzyme Research Center, Framingham, USA) or activin-A (10 ng/mL, PeproTech EC Ltd, London, UK) for the time periods indicated in the figures. The N2B27 media in glioblastoma cells were changed prior to TGFβ1 treatment. The LY2157299 TβRI kinase inhibitor (Sigma-Aldrich AB, Stockholm, Sweden) was administered to cells one hour before TGFβ1 addition at a final concentration of 2.5 nM. The MAP kinase kinase inhibitor (MEKi; PD184352, Sigma-Aldrich AB, Stockholm, Sweden) was added at a concentration of 0.5 µM, the Jun N-terminal kinase inhibitor (JNKi; SP600125, Calbiochem-Merck, Stockholm, Sweden) and the p38 MAP-kinase inhibitor (P38i; SB203580, Calbiochem-Merck, Stockholm, Sweden) were added at a concentration of 10 µM. Protein synthesis was blocked by cycloheximide (CHX; C1988, Sigma-Aldrich AB, Stockholm, Sweden), administered to the cells 1 h before TGFβ treatment at a final concentration of 40 µg/ml. Dimethyl-sulfoxide (DMSO) served as vehicle for all chemicals.

### **Cloning of human*LINC00707***

The human *LINC00707* RNA (NR_038291.1) was amplified by RT-PCR using cDNA from immortalized human mammary epithelial MCF-10A cells and specific cloning primers. Total RNA from MCF-10A cells was converted to cDNA using the PrimeScript 1st strand cDNA synthesis kit (Takara Bio Europe, Saint-Germain-en-Laye, France), following the instructions by the manufacturer. The *LINC00707* insert DNA, consisting of 3,087 bp, was ligated to the pcDNA3 vector between the *Kpn*1 and *Xho*I restriction enzyme sites (Additional file 5, Fig. S1A). The pcDNA3-LINC00707 construct was verified by sequencing. The cloning primers were: Fw, 5’-AAAAGGTACCAAAATAAAGCAGGCAGAGGAACGTGGAAGGACT-3’, Rev, 5’-TTTTTCTCGAGTTTTTTTTTTTTTTTTTTGGCTCAACAGTCAAAGACAGGTTTATTTTGGA-3’. The sequencing primers were: pcDNA3CMV promoter, Fw, 5’-GTCAATGGGAGTTTGTTTTGG-3’; pcDNA3 BGH polyA R1149, Rev, 5´-GCATCGCATTGTCTGAGTAG-3’; *LINC00707* seqF654, 5´-GTCAGACTGAGCACCACTCCC-3’; *LINC00707* seq1431, 5´-GAGCTAGAAGTTAGAAGGTCTAGG-3´; *LINC00707* seq2147, 5´-CTTCCCTTCAGCTGCTGG-3’.

### Transient, stable transfections and lentiviral infections

Transient transfections with siRNAs and plasmids were performed using the *Trans*IT-X2® reagent (Mirus Bio, Madison, WI, USA. Ref: MIR 6000) in PC3U, U3031MG and U3034MG cells according to the manufacturer’s protocol. HaCaT cells were transfected with siLentfect (Bio-Rad, Hercules, CA, USA, siLentFect™ Lipid Reagent, Ref:170–3362) for siRNAs or with Lipofectamine 3000 (Invitrogen, ThermoFischer Scientific, Uppsala, Sweden) for plasmids, according to the manufacturer’s protocol. Twenty-four hours after transfection, the growth medium was changed to starvation medium (serum-free DMEM or RPMI). Cells were transfected with siRNAs targeting mRNAs at a final concentration of 25 nM, or with plasmid at a final concentration of 1 µg/ml for 24 h and were then treated with TGFβ1 or vehicle for the indicated time periods. Stable transfection of U3031MG and U3034MG cells with control pcDNA3 or pCDNA3-LINC00707 vectors was performed as previously described [19], using G-418 selection and single cell cloning to generate the clones U3031MG- and U3034MG-pcDNA, U3031MG- and U3034MG-pcLINC1, as well as U3031MG- and U3034MG-pcLINC2, as presented in the figures. Plasmids and siRNAs used for transfections are shown in Additional file 1-Table S1.

The infection of HaCaT cells with short hairpin RNAs (shRNAs) targeting *LINC00707* was performed using MISSION pLKO.1-puro lentiviral constructs. Cells were infected at a final multiplicity of infection (MOI) of 10. The efficiency of transduction and the evaluation of the optimal MOI for the lentiviral infections were confirmed using MISSION pLKO.1-puro-CMV-TurboGFP positive control lentivirus (SHC003V, Sigma-Aldrich AB, Stockholm, Sweden). For control infection, a MISSION non-mammalian shRNA control (SHC002V, Sigma-Aldrich AB, Stockholm, Sweden) was used. Three days after infection, the cells were treated with 2 µg/ml puromycin for two weeks. Then, the efficiency of *LINC00707* silencing was confirmed by RT-qPCR analysis. The shRNA sequences targeting the human *LINC00707* lncRNA were: shLINC00707#2, 5´-ACTGCCACCGTGATTTATTTA-3’ and shLINC00707#3, 5´-TGCTGGCTTGAATATGTTAAT-3’.

### Luciferase assay

PC3U cells were transiently transfected using *Trans*IT-X2® reagent with TGFβ/Smad-responsive promoter-reporter pCAGA_12_-MLP-luc for 24 h prior to stimulation with TGFβ. pCMV-β-gal was co-transfected as control for normalization. Additional constructs or siRNAs were included in the transfections according to the figures. All cells were lysed in lysis buffer containing 5 mM Tris-phosphate buffer, pH 7.8, 2 mM dithiothreitol (DTT), 2 mM trans-1,2-diaminocyclohexane-*N*,*N*,*N′*,*N′*-tetra-acetic acid, 5% glycerol, and 1% Triton X-100. The β-galactosidase assay was performed by mixing the cell lysate with 100 mM sodium phosphate, pH 7.3, 1 mM MgCl_2_, 50 mM β-mercaptoethanol, and 0.67 mg/ml of *o*-nitrophenyl β-d-galactopyranoside, and the absorbance was monitored at 420 nm. Luciferase reporter assays were performed with the enhanced luciferase assay kit from BD PharMingen, Inc. (BD Biosciences, Stockholm, Sweden), according to the protocol of the manufacturer. Normalized promoter activity data are plotted in bar graphs that represent average values from triplicate determinations with standard deviations. Each independent experiment was repeated at least three times.

### HTA2 Affymetrix and LncPath human EMT pathway microarray analysis

Transcriptomic analysis in U3031MG cells stimulated or not with TGFβ1 for 24 h was performed by an HTA2 Affymetrix Platform array (ThermoFischer Scientific, Uppsala, Sweden); the expression profiles have been deposited to Array Express with accession number E-MTAB-9076. Primary data from this analysis have been presented in Ref [19]. For each condition, triplicates were analyzed by the Swegene centre for Integrative Biology at Lund University (SCIBLU). Transcriptome Analysis Console (TAC) v.4.0.2 was used to perform differential gene expression analysis. Adjusted *p*-values (p-adj) for multiple testing, using Benjamini-Hochberg to estimate the false discovery rate (FDR), were calculated for final estimation of differential expression (DE) significance, genes with FDR < 0.1 and FC > 2 or <-2 were selected.

Total RNA from HaCaT cells, treated with TGFβ1 for 24 h or untreated (control) cells was isolated using the RNeasy kit (Qiagen AB, Sollentuna, Sweden) and subjected to microarray analysis using the LncPath™ Human Epithelial to Mesenchymal Transition (EMT) Array (Arraystar Inc, Rockville, MD, USA). The detailed analysis has been published previously [13]. Subsequent data processing was performed using the R software package (https://www.r-project.org/). Differentially expressed LncRNAs/mRNAs with statistical significance between two samples were identified through Volcano Plot filtering. Differentially expressed LncRNAs/mRNAs between two samples were identified through Fold Change filtering [13]. Primary data from this analysis have been presented in [13].

### AmpliSeq Transcriptome analysis

Total RNA from HaCaT cells, transfected with a non-targeting shRNA (shC) and treated with TGFβ1 for 24 h (shC + TGFβ) or stably transfected with two different shRNAs targeting *LINC00707* (shLINC00707#2 and shLINC00707#3), was isolated using the NucleoSpin RNA Plus Kit (Macherey-Nagel, AH Diagnostics, Solna, Sweden). Total RNA (50 ng) was reverse-transcribed to cDNA using Ion AmpliSeq™ Transcriptome Human Gene Expression Kit Preparation protocol (Revision A.0, Life Technologies Ltd, Paisley, UK). The acquired cDNA was amplified using Ion AmpliSeq™ Transcriptome Human Gene Expression core panel (Life Technologies Ltd, Paisley, UK) and the primer sequences were partially digested. Adaptors (Ion P1 Adapter and Ion Xpress™ Barcode Adapter, Life Technologies Ltd, Paisley, UK) were ligated to the amplicons. Adaptor‐ligated amplicons were purified using Agencourt® AMPure® XP reagent (Beckman, Coulter Inc., Brea, CA, USA) and eluted in amplification mix (Platinum® PCR SuperMix High Fidelity and Library Amplification Primer Mix, Life Technologies Ltd, Paisley, UK) and amplified. Size‐selection and purification was conducted using Agencourt® AMPure® XP reagent (Beckman, Coulter Inc., Brea, CA, USA). The amplicons were quantified using the Fragment Analyzer™ instrument (Advanced Analytical Technologies, INC., Ankeny, IA, USA) with DNF‐474 High Sensitivity NGS Fragment Analysis Kit (Advanced Analytical Technologies, INC., Ankeny, IA, USA). Samples were then pooled (six or less per pool), followed by emulsion PCR on either the Ion OneTouch™ two System using the Ion PI™ Hi‐Q™ OT2 Kit (Life Technologies Ltd, Paisley, UK), or on the Ion Chef™ System using the Ion PI Hi‐Q Chef Kit (Life Technologies Ltd, Paisley, UK). The pooled samples were loaded on Ion PI™ v3 chips and sequenced on the Ion Proton™ System using the Ion PI™ Hi‐Q Sequencing 200 Kit chemistry (200 bp read length, Life Technologies Ltd, Paisley, UK). Acquired reads were aligned to the hg19 AmpliSeq Transcriptome ERCC v1 using the Torrent Mapping and Alignment Program (tmap) with default settings. Sequence reads were analyzed by AmpliSeqRNA analysis plugin, v4.2.1, in the Torrent Suite Software (Life Technologies Ltd, Paisley, UK), counting the number of sequences obtained for all cDNA amplicons. The resulting counts represent gene expression levels for over 20,800 genes, which were merged into a table, used for differential gene expression analysis with the R/Bioconductor package EdgeR [20] (http://www.bioconductor.org/) using standard parameters. Adjusted *p*-values (padj) for multiple testing, using Benjamini-Hochberg analysis to estimate the FDR, were calculated for final estimation of differential expression significance, followed by functional enrichment using the R package clusterProfiler [21] (www.bioconductor.org). All primary data from this analysis have been deposited in ArrayExpress and are available under accession number: E-MTAB-12,980 (https://www.ebi.ac.uk/biostudies/arrayexpress/studies/E-MTAB-12980).

### RNA interaction and ChIP enrichment analysis

In order to identify potential proteins that interact with *LINC00707*, we used the RNAinter (http://www.rnainter.org/) database using the standard options of the RNAinter software. A cut-off value of 0.2 was selected based on the score of previously characterized *LINC00707*-interacting proteins (e.g. HuR).

Genes commonly up-regulated in HaCaT cells with stable *LINC00707* silencing (shLINC00707#2 and shLINC00707#3) with an FDR < 0.1 and a logFC<-2, were further selected and analyzed using ChIP Enrichment analysis (ChEA) based on the Enrichr program (http://amp.pharm.mssm.edu/Enrichr/), version 2016 (ChEA 2016) in order to identify transcription factor binding sites on the promoters of the selected genes.

### Chromatin immunoprecipitation

PC3U cells at 80% confluency in 15-cm dishes were treated or not with TGFβ1 (5 ng/ml) for 24 h and then crosslinked in 1% formaldehyde for 10 min at room temperature. The crosslinking was quenched by addition of 125 mM glycine for 5 min at room temperature. The cells were then washed and scraped in ice-cold PBS and centrifuged (4,000 rpm, 5 min). The cell pellets were stored at minus 80 °C until further use. The cell pellets were lysed in ChIP lysis buffer (50 mM Tris-HCl, pH 8, 10 mM EDTA, 1% SDS), supplemented with protease inhibitors (Roche Diagnostics Scandinavia AB, Bromma, Sweden). The lysates were subjected to sonication for 10 min in a water bath Diagenode Bioruptor sonicator (Diagenode, Bionordika, Stockholm, Sweden), with 30 s pulses for 10 min, to shear the chromatin to ~ 500 bp long DNA fragments. After sonication, the lysates were centrifuged at 14,000 rpm for 10 min at 4 °C. The supernatant (90% of the volume) was diluted 10 times in ChIP dilution buffer (20 mM Tris-HCl, pH 8.0, 2 mM EDTA, 1% Triton X-100, 150 mM NaCl), supplemented with protease inhibitors (cOmplete EDTA-free protease inhibitor cocktail, Roche Diagnostics Scandinavia AB, Bromma, Sweden) and used for immunoprecipitation. The remaining 10% of the lysate was used as an input. KLF6 antibody (10 µg, 39-6900, ThermoFischer Scientific, Uppsala, Sweden), Smad2/3 antibody (10 µg, 610,843, BD Biosciences-Europe, Stockholm, Sweden) or normal mouse IgG (Millipore/Merck, Stockholm, Sweden) were coupled with sheep anti-mouse IgG dynabeads M-280 (Invitrogen, ThermoFischer Scientific, Uppsala, Sweden), in 0.5% BSA (IgG-free)/PBS solution (with overnight end-over-end rotation at 4 °C) and used for the ChIP assay. The lysates with the antibody-bead complexes were incubated at 4 °C overnight with end-over-end rotation. The precipitated complexes were washed 5 times in RIPA buffer (50 mM HEPES-KOH, pH 7.0, 0.5 M LiCl, 1 mM EDTA, 0.7% DOC, 1% NP-40), with an additional final wash in TE buffer (50 mM Tris-HCl, pH 8.0, 10 mM EDTA). Then, elution buffer was added to precipitated complexes, as well as to input samples, and samples were subjected to reverse crosslinking overnight at 65 °C. After that, the immunoprecipitated DNA was purified, using the QIAquick PCR Purification kit, according to the protocol by the manufacturer (QIAGEN AB, Sollentuna, Sweden). A complete list of the oligonucleotides used for ChIP-qPCR assays is shown in Additional file 2-Table S2.

### Real-time RT-PCR

Total RNA from HaCaT, PC3U, MCF-10 A, MDA-MB-231, A549, H1299, U3031MG, U3034MG and U2987MG cells was extracted using the NucleoSpin RNA kit (Macherey-Nagel, AH Diagnostics, Solna, Sweden) or the RNeasy kit (Qiagen AB, Sollentuna, Sweden) according to the protocol of the manufacturer. cDNA was synthesized using the iScript cDNA synthesis kit from Bio-Rad Laboratories AB, Solna, Sweden. Real-time PCR was done in triplicate using the qPCRBIO SyGreen 2×Master Mix (PCR Biosystems, London, UK) and gene-specific primers listed in Additional file 2-TableS2. Gene expression levels were determined by the comparative C_t_ method and using *HPRT1* (hypoxanthine phospho-ribosyl transferase 1) or *GAPDH* (glyceraldehyde 3´-phosphate dehydrogenase) as reference. Normalized mRNA expression levels are plotted in bar graphs that represent average values from triplicate determinations with standard deviations. Each independent experiment was repeated at least three times.

### Separation of nuclear and cytoplasmic fractions and immunoblotting

For RNA extraction, nuclear and cytoplasmic fractions of HaCaT, PC3U or U3034MG cells were separated using the PARIS kit (AM1921; ThermoFischer Scientific, Uppsala, Sweden) followed by RNA extraction and storage at -70^o^C, according to the manufacturer’s protocol. Fractionated RNA was then analyzed by RT-qPCR.

For protein extraction, nuclear and cytoplasmic fractions of PC3U and U3034MG cells were separated after transfection with control or *LINC00707* siRNAs or plasmids and treatment or not with TGFβ1 (5 ng/ml) for 1 h. Cells were rinsed with PBS twice, scraped in 1 ml of fresh PBS and centrifuged at 4^o^C for 5 min at 450×g. The pellet was resuspended in 200 µl of lysis buffer (50 mM Tris-HCl pH7.5, 10 mM MgCl_2_, 15 mM CaCl_2_, 1.5 M sucrose) complemented with 1% of protease inhibitor and 1% of 0.1 M of DTT. Cells were incubated on ice of 15 min and 12 µL of 10% IGEPAL CA-630 were added before agitation and centrifugation for 30 s at 11,000×g. The supernatant represents the cytoplasmic fraction. The pellet was resuspended in 50 µl of nuclear extraction buffer (20 mM HEPES pH7.9, 1.5 mM MgCl_2_, 0.42 M NaCl, 0.2 mM EDTA, 25% glycerol) complemented with 1% of protease inhibitor and 1% of 0.1 M DTT and agitated for 20 min at 4^o^C. Nuclear fraction is obtained by centrifugation for 5 min at 20,000×g at 4^o^C. For total protein analysis from transfected and/or stimulated cells, proteins were extracted in a nonidet P-40 (NP-40)-containing lysis buffer (20 mM Tris-HCl, pH 8.0, 1% NP-40, 150 mM NaCl, 2 mM EDTA, and complete protease inhibitor mixture from Roche Diagnostics Scandinavia AB, Bromma, Sweden). Proteins were then quantified and subjected to SDS-PAGE.

The resolved proteins were transferred to nitrocellulose using a wet transfer unit (Bio-Rad Laboratories AB, Solna, Sweden). The efficiency of immunoblotting and equal loading of proteins was verified by staining of the nitrocellulose membrane with 0.1% (w/v) Ponceau S in acetic acid. Upon incubation with primary antibodies and horseradish peroxidase-conjugated secondary antibodies, enhanced chemiluminescence assays were performed using the Millipore kit (Merck-Millipore, Stockholm, Sweden). The antibodies used were: anti-Smad2 (5339 S, Cell Signaling Technology, Danvers, MA, USA), anti-Smad3 (9523 S, Cell Signaling Technology, Danvers, MA, USA), anti-pSmad2 (home-made, Ludwig Cancer Research-Uppsala Branch, Sweden), anti-pSmad3 (9520 S, Cell Signaling Technology, Danvers, MA, USA), anti-PARP-1 (5625, Cell signaling Technology, Danvers, MA, USA), anti-ALIX/HP95 (HPA011905, Sigma-Aldrich, Stockholm, Sweden), anti-GAPDH (AM4300, ThermoFischer Scientific, Uppsala, Sweden), anti-PAI1 (ab66705, Abcam, Cambridge, UK), anti-fibronectin (F3648, Sigma-Aldrich AB, Stockholm, Sweden), anti-N-cadherin (3195 S, Cell Signaling Technology, Danvers, MA, USA), anti-ZEB1 (HPA027524, Sigma-Aldrich AB, Stockholm, Sweden), anti-KLF6 (39-6900, ThermoFischer Scientific, Uppsala, Sweden), anti-vimentin (5741 S, Cell Signaling Technology, Danvers, MA, USA), anti-cyclinE (4129T, Cell signaling Technology, Dancers, MA, USA), and anti-CDKN1A/p21 (610,233, BD Biosciences-Europe, Stockholm, Sweden).

Quantification of protein signals by densitometry was performed using National Institutes of Health ImageJ software. Band densities for protein of interest were normalized to that of the band for GAPDH or HP95 in the same sample.

### RNA-FISH

In situ hybridization of *LINC00707* RNA was performed according to the Stellaris RNA-FISH protocol for adherent cells (Biosearch Technologies, Bio-Mediator Ky, Vantaa, Finland), and as described earlier [13]. The design of the specific probes for detecting *LINC00707* was performed following the Stellaris RNA FISH Probe designer. The *LINC00707* FISH probes were coupled to the CAL Fluor® Red 590 Dye. The primary antibody (anti-Lamin B1, ab16048, Abcam, Cambridge, UK) was added together with specific *LINC00707* probes in hybridization buffer and incubated with HaCaT cells at 37^o^C for 16 h. Then, a secondary antibody (donkey anti-rabbit Alexa Fluor 4888, A-21,206, Invitrogen, ThermoFischer Scientific, Uppsala, Sweden) was added in washing buffer A and incubated with the cells for 30 min at room temperature. Next, washing buffer A containing 4′,6-diamidino-2-phenylindole (DAPI) and secondary antibody was added to the cells for 30 min at room temperature, and finally samples were mounted on Fluoromount-G (ThermoFischer Scientific, Uppsala, Sweden) and examined on a Nikon Eclipse 90i fluorescence microscope (Nikon Corp., Tokyo, Japan). Photography was done at ambient temperature in the absence of immersion oil, using the Nikon 20×/0.75 Plan-Apo objective lens. Images were acquired with a Nikon Digital Sight DS-Qi1Mc CCD camera and the acquisition software NIS-Elements AR 3.2 using the same exposure time and without additional processing. The figures present cropped parts of the original images without additional processing.

### RNAscope analysis and immunohistochemistry

The localization of *LINC00707* was assessed using the RNAscope Multiplex Fluorescent Reagent Kit v2 (Advanced Cell Diagnosis, Newark, CA, USA) in adherent PC3U cells transfected with control or *LINC00707* plasmids, U3034MG cells treated or not with TGFβ1 (5 ng/ml) for 24 h and in glioblastoma tumor samples. Adherent cells were prepared by fixation with 3.7% formaldehyde in PBS, permeabilized with 0.5% Triton X-100, treatment with hydrogen peroxide for 10 min at room temperature (provided by the kit) and protease III for 10 min at room temperature (provided by the kit). The rest of the protocol was performed as described by the manufacturer. Dehydrated tumor samples were first deparaffinized in successive baths of xylene for 5 min, 100% ethanol and 70% ethanol for 2 min. The staining was performed as described by the manufacturer. Slides were boiled in a steamer (Braun - FS3000WH, Braun GmbH, Germany) and the signal was visualized using the fluorophore Opal 690 (Akoya Biosciences, Marlborough, USA), diluted 1:1,500. Slides were washed twice with TBS-T and treated with serum-free blocking solution ready to use (Agilent Technologies, X0909, Santa Clara, CA, USA) for 10 min at room temperature. The primary antibody (anti-pSmad2 home-made, Ludwig Cancer Research-Uppsala Branch, Sweden) diluted at 1:200 in buffer (Agilent Technologies, S0908, Santa Clara, CA, USA) was applied and samples were incubated at 4 °C overnight. Secondary antibody coupled with Alexa Fluor 594 (ThermoFischer Scientific, Uppsala, Sweden) complemented with DAPI 1:1,000 (Sigma-Aldrich Sweden AB, Stockholm, Sweden) was incubated for 1 h at room temperature and then washed three times with TBS-T. Pictures were taken on a Nikon Eclipse 90i fluorescence microscope (Nikon Corp., Tokyo, Japan). Photography was done at ambient temperature in the absence of immersion oil, using the Nikon 20×/0.75 Plan-Apo objective lens. Images were acquired with a Nikon Digital Sight DS-Qi1Mc CCD camera and the acquisition software NIS-Elements AR 3.2 using the same exposure time and without additional processing. The figures present cropped parts of the original images without additional processing.

### RNA immunoprecipitation

PC3U cells (2 × 10^7^) were harvested and crosslinked using 1% formaldehyde for 10 min at room temperature followed by quenching using 0.125 M of glycine for 5 min. The crosslinked cells were centrifuged at 1,000×*g* at 4 °C for 10 min and the pellet was resuspended in 600 µl of RIPA lysis buffer (150 mM NaCl, 50 mM Tris HCl pH7, 0.2% SDS, 0.5% sodium deoxycholate, 1% NP-40, 50 units/ml RNasin) and sonicated using a Bioruptor for 10 min (30 s on, 30 s off). Insoluble material was removed by centrifugation at 13,000×*g* at 4 °C for 10 min. Sonicated material enriched in the range of 100–500 bp was divided into 3 groups: 100 µl were used as an input control, 250 µl were incubated with 2 µg of the respective mouse antibodies against Smad2/3 (610,843, BD Biosciences-Europe, Stockholm, Sweden) or HuR (39–0600, ThermoFischer Scientific, Uppsala, Sweden) and 250 µl were incubated with 2 µg of IgG control for 2.5 h at 4 °C. The antibodies were before-hand coupled with sheep anti-mouse IgG dynabeads M-280 (Invitrogen, ThermoFischer Scientific, Uppsala, Sweden) for 3 h at 4 °C. The complexes bound to beads were obtained by magnetic precipitation followed by one wash each of low salt buffer (0.1% SDS, 0.5% NP-40, 1×PBS, 50 units/ml RNasin) and high salt buffer (0.1% SDS, 0.5% NP-40, 5×PBS, 50 units/ml RNasin). The immune-precipitated material was resuspended in 50 µl of RNA extraction buffer (100 mM NaCl, 10 mM Tris-HCL pH7.0, 0.1 mM EDTA, 0.5% NP-40, 10 mg/ml Proteinase K) and eluted from the beads at 65 °C for 45 min followed by 95 °C incubation for 15 min. The samples were kept in 1 mL trizol at -20 °C until the RNA was purified by phenol–chloroform extraction. After RNA extraction, the pellet was resuspended in 10 µl of water and subjected to reverse-transcription using the iScript cDNA synthesis kit from Bio-Rad Laboratories AB, Solna, Sweden. RT-qPCR was done as previously described [13] in triplicate.

### ChOP assay

Oligonucleotide-based RNA precipitation was performed using a modification of the chromatin oligo-affinity precipitation (ChOP) protocol [22]. Two billion PC3U cells were collected, washed twice with PBS at room temperature, and crosslinked in 1% v/v formaldehyde/PBS by rotating the cells for 10 min at room temperature. Upon quenching the reaction using 125 mM glycine at room temperature and gentle rotation and washing twice with ice-cold PBS, the fixed cells were centrifuged at 4 ^o^C and 2,000×g for 5 min. Cell pellets were lysed in 50 mM Tris HCl pH 8, 10 mM EDTA, 0.1% SDS, supplemented with protease inhibitors (cOmplete EDTA-free protease inhibitor cocktail, Roche Diagnostics Scandinavia AB, Bromma, Sweden) and 100 units/ml RNase inhibitor (Thermo Fisher Scientific, Uppsala, Sweden), on ice for 20 min. Cell lysates were first sonicated at high pulse during 20 cycles (Bioruptor sonicator; Diagenode, Bionordika, Stockholm, Sweden) and then incubated with 20 µL of streptavidin-coupled agarose beads (Sigma-Aldrich Sweden AB, Stockholm, Sweden) for 30 min for pre-clearing. After centrifugation, a volume of 50 µL of total cell lysate was collected and diluted in 2× Laemmli buffer containing 10% dithiothreitol, as input. Pre-cleared lysates were then incubated overnight via gentle rotation at 4 °C with 100 µg/ml yeast tRNA, 100 µg/ml salmon sperm DNA and a final concentration of 10 µM of a pool of 7 distinct, *LINC00707*-specific, biotinylated oligonucleotides spanning the entire 3,087 nt long transcript that could capture *LINC00707* and putative associated proteins. Biotinylated oligonucleotides specific for the *E. coli LacZ* transcript served as negative control. RNA-protein complexes were collected by rotating incubation with streptavidin-coupled agarose beads (Sigma-Aldrich Sweden AB, Stockholm, Sweden) for 2 h at 4 °C, washing with low-salt buffer (20 mM Tris-HCl, pH 7.9, 150 mM NaCl, 2 mM EDTA, 0.1% SDS, 1% Triton X-100, 0.5 mM PMSF and 50 units/ml RNase inhibitor) three times for 5 min at 4 °C, followed by another two washes for 5 min each at 4 °C with high-salt buffer (low-salt buffer containing 500 mM NaCl), two final washes with PBS at room temperature and elution of the nucleoprotein complexes from the beads by vigorous agitation in 100 µl of 1× Laemmli buffer containing 10% dithiothreitol at 95 °C for 15 min. The purified eluents were subjected to SDS-PAGE and immunoblotting. The specific and control oligonucleotides are listed in Additional file 2-Table S2.

### Cell proliferation assay

Cell proliferation was assessed using the Click-IT Plus Edu kit (C10640, ThermoFischer Scientific, Uppsala, Sweden). PC3U cells (5,000 cells/well) were seeded in multi-well 8 plates (Falcon, ThermoFischer Scientific, Uppsala, Sweden), transfected with control, *LINC00707* siRNAs or plasmids, and treated or not with TGFβ1 (5 ng/ml) for 72 h. Cells were labeled with 10 µM of 5 ´-ethynyl-2 ´- deoxyuridine (Edu) for 3 h and fixed with 3.7% formaldehyde in PBS for 15 min at room temperature. Cells were then washed with 3% BSA in PBS before permeabilization with 0.5% Triton X-100 in PBS for 20 min at room temperature. Cells were washed again with 3% BSA in PBS before detection of Edu by Click-IT Plus reaction cocktail prepared as described by the manufacturer and incubated for 30 min at room temperature protected from light. Cells were washed once with 3% BSA in PBS, once with PBS and incubated with 5 µg/ml of Hoechst 33,342 provided by the kit for 30 min at room temperature protected from light. For quantification, 10 pictures of each cell culture well of three independent repeats were taken at 20× magnification on a Nikon Eclipse 90i fluorescence microscope (Nikon Corp., Tokyo, Japan), and Edu-positive cells were counted using the ImageJ software (National Institutes of Health, Bethesda, MD, USA).

### Cell invasion assay

The invasion assay was designed using transwell plates with 6.5 mm diameter and 8 μm pore filters (351,152, Corning Costar, NY, USA or 140,629, ThermoFischer Scientific, Uppsala, Sweden). Inserts were coated with 100 µg/ml collagen (5005, Advanced BioMatrix Inc., San Diego, CA, USA) or 10 µg/ml laminin and incubated overnight at 37 °C. The U3031MG and U3034MG cells (5 × 10^4^), control and overexpressing *LINC00707* or transfected with control and *LINC00707* siRNAs were seeded in the upper chamber coated with laminin, while PC3U (5 × 10^4^) cells transfected with control, *LINC00707* siRNAs or plasmids were seeded in the upper chamber coated with collagen. For all cell lines, cells were seeded in the upper chamber in serum-free DMEM complemented or not with TGFβ1 (5 ng/ml), and DMEM/6% FBS was placed in the lower chamber. Following 15 h, inserts were fixed with ice-cold methanol. Nuclei were stained with DAPI 1:1,000 (Sigma-Aldrich Sweden AB, Stockholm, Sweden). Invasion of the cells was measured by counting the nuclei of the cells that migrated through the filter pores towards the chemoattractant (6% FBS) and invaded into the respective matrix. For quantification, 10 pictures of each insert were taken at 20× magnification using a Nikon Eclipse 90i fluorescence microscope (Nikon Corp., Tokyo, Japan), and nuclei were counted using the ImageJ software (National Institutes of Health, Bethesda, MD, USA). Results are expressed in number of cells per picture.

### ELISA

TGFβ1 and TGFβ2 were measured in conditioned medium of PC3U cells using human TGFβ1 Quantikine ELISA kit (DB100B, R&D Systems Inc., Minneapolis, MN, USA) and TGFβ2 Quantikine ELISA kit (DB250, R&D Systems Inc., Minneapolis, MN, USA), respectively, according to the manufacturer’s instructions.

### Statistical analysis

All figure legends and each detailed method present the biological and technical replicates and the assessment of statistical significance. The statistical method used was based on sample content and variation within each data-set; similar variance existed between the compared group populations. Accordingly, two-group comparisons were performed using two-tailed unpaired Student´s t-test and multiple group comparisons were performed using ANOVA with Bonferroni correction. Statistical significance is represented by *p*-values *p ≤ 0.05, **p ≤ 0.01, ***p ≤ 0.001.

Statistical analysis was performed using the software Graphpad Prism v.7 (GraphPad Software, San Diego, CA, USA).

## Results

### **TGFβ down-regulates*LINC00707***

**Fig. 1:**
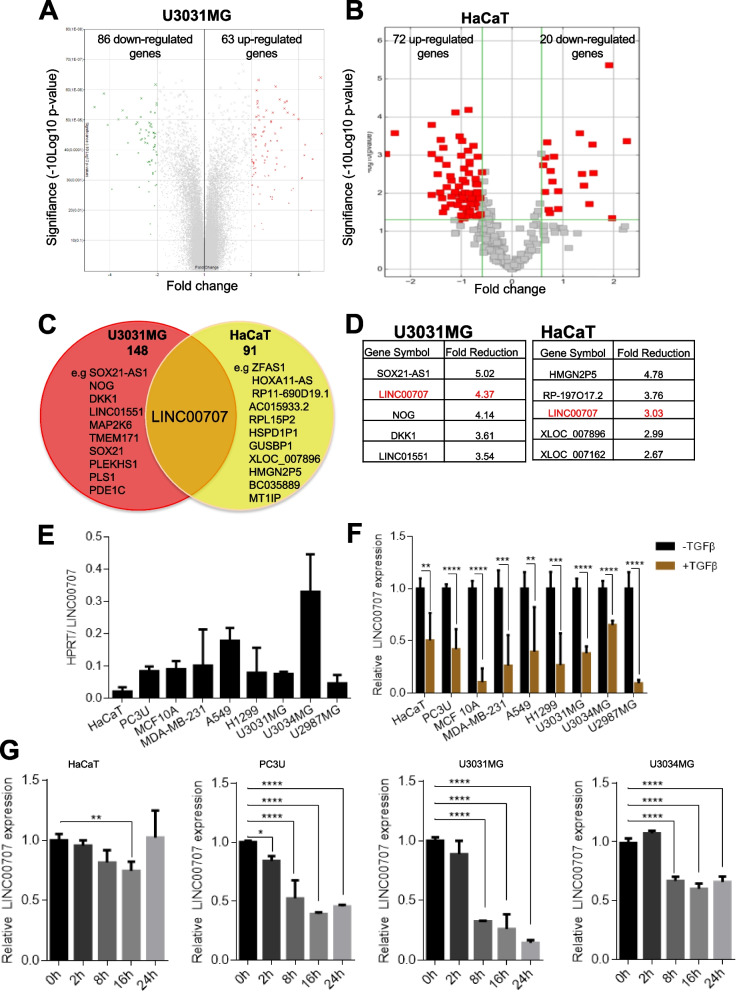
*LINC00707* is down-regulated by TGFβ signaling. **A**,** B** Volcano plots of differentially-regulated genes, in response to TGFβ treatment for 24 h, in U3031MG (A) and HaCaT (B) cells. Fold-change threshold was set to + 2 and − 2; U3031MG up-regulated genes are shown in red, down-regulated genes are shown in green (A). Up-regulated and down-regulated genes are shown in red (B). **C** Venn diagram illustrating *LINC00707* as a common down-regulated gene in response to TGFβ treatment for 24 h between U3031MG and HaCaT cells. **D** Lists of the top-5 down-regulated genes in response to TGFβ treatment for 24 h, in U3031MG and HaCaT cells. **E**,** F** RT-qPCR for determination of the basal *LINC00707* expression (E) and its expression after TGFβ stimulation for 24 h or not (F), in HaCaT, PC3U, MCF-10A, MDA-MB-231, A549, H1299, U3031MG, U3034MG and U2987MG cells. **G** RT-qPCR for determination of *LINC00707* expression in HaCaT, PC3U, U3031MG and U3034MG cells in response to TGFβ treatment for the indicated time periods. RT-qPCR graphs present data from (*n* = 3) biological repeats, each with three technical repeats, as means with SEM. Statistical significance of the variables was assessed by Student´s t-test (panel F) or ANOVA test with Bonferroni correction (panel G). No statistical analysis was performed on panel E due to the unique set of data

We identified TGFβ-regulated lncRNAs in normal and cancerous cellular systems, by combining two transcriptomic analyses, in two well-established and highly TGFβ-responsive cell models, one in patient-derived glioblastoma cells (U3031MG), and another in immortalized human keratinocytes (HaCaT). Affymetrix microarray analysis of U3031MG cells [19] profiled expression of 25,000 genes in total (Fig. 1A). LncPath Human EMT Pathway Microarray analysis of HaCaT cells [13], profiled expression of 773 lncRNAs and 219 mRNAs in total (Fig. 1B). By comparing the TGFβ-regulated genes in the two profiles, we identified *LINC00707* as a common down-regulated gene in response to TGFβ stimulation (Fig. 1C, D). We validated the transcriptomic results in several human cell models (HaCaT, prostate carcinoma (PC3U), breast epithelial (MCF-10A) and breast carcinoma (MDA-MB-231), lung adenocarcinoma (A549, H1299), and glioblastoma (U3031MG, U3034MG, U2987MG)), by RT-qPCR (Fig. 1E, F). *LINC00707* was expressed at different basal levels (Fig. 1E) and TGFβ decreased *LINC00707* expression in all cell models (Fig. 1F). *LINC00707* down-regulation in response to TGFβ was analyzed deeper in HaCaT, PC3U, U3031MG and U3034MG cells and was time-dependent, with maximal decrease observed at late time points of stimulation (Fig. 1G). We also confirmed that *LINC00707* is specifically regulated by TGFβ as the simulation of HaCaT (Additional file 5, Fig S1 A) and PC3U (Additional file 5, Fig S1B) cells by other ligands of the TGFβ family failed to down-regulate *LINC00707*. These analyses established that *LINC00707* expression is modulated by TGFβ stimulation.

### ***LINC00707*exhibits cytoplasmic localization**

**Fig. 2:**
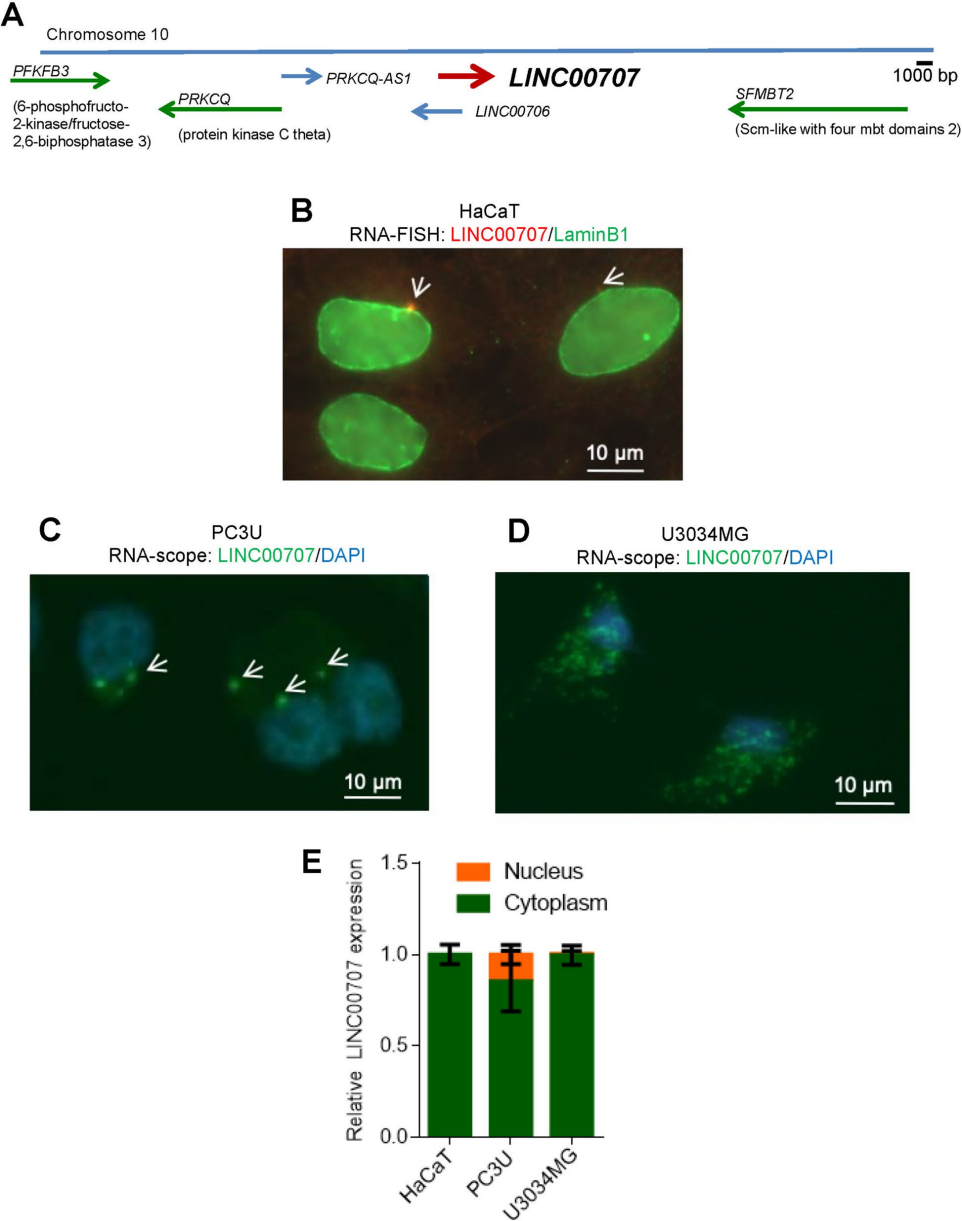
*LINC00707* resides mostly in the cytoplasm. **A** Schematic representation of the genomic location of the *LINC00707* gene, together with its neighboring genes at chromosome 10. Arrows point to gene transcripts along with the direction of their transcription. **B** RNA-FISH for *LINC00707* combined with immunofluorescence for lamin-B1 in HaCaT cells. The arrows depict specific *LINC00707* RNA spots. Magnification bars (10 μm). **C**,** D** RNAscope for transiently overexpressed *LINC00707* in PC3U cells (C) and for endogenous *LINC00707* in U3034MG cells (D). The arrows depict specific *LINC00707* RNA spots. Magnification bars (10 μm). **E** RT-qPCR for determination of *LINC00707* in nuclear and cytoplasmic fractions of HaCaT, PC3U and U3034MG lysates. The graphs present data from (*n* = 2) biological repeats, each with three technical repeats, as percentage of total (nuclear plus cytoplasmic) RNA expression with SEM

The human gene encoding *LINC00707* maps as an intergenic lncRNA on chromosome 10 (10p14), between the protein-coding *PRKCQ* and *SFMBT2* genes, and contains 5 exons and 4 introns (Fig. 2A). NCBI classifies *LINC00707* as a long lncRNA that is experimentally validated and spliced to a single 3,087 nt RefSeq transcript (NR_038291.1).

By performing concurrent RNA-FISH using *LINC00707* antisense probes and immunostaining for the nuclear envelope protein lamin-B1, we observed mostly cytoplasmic RNA signals proximal to the nuclear envelope of HaCaT cells (Fig. 2B), implying export of *LINC00707* to the cytoplasm after transcription. As a complementary approach, the RNAscope method was used. In order to visualize *LINC00707* independent from its variable endogenous level of expression, we cloned the human *LINC00707* cDNA using a library available to our laboratory, generated from immortalized human breast epithelial MCF-10A cells (Additional file 5, Fig. S2A). Analysis by RT-qPCR confirmed an efficient overexpression of pc*LINC00707* in PC3U cells (Additional file 5, Fig. S2B) allowing us to reach a sufficient level for visualization by RNAscope (Fig. 2C). Endogenous *LINC00707* was detected in U3034MG by RNAscope (Fig. 2D). All cell lines confirmed the cytoplasmic localization of endogenous and overexpressed *LINC00707*. After TGFβ stimulation, *LINC00707* was down-regulated and became barely detectable by RNAscope (Additional file 5, Fig. S2C, shown for U3034MG cells). We corroborated the RNA localization studies with RT-qPCR assays that examined *LINC00707* expression in nuclear and cytoplasmic fractions of HaCaT, PC3U and U3034MG cells, verifying higher enrichment of *LINC00707* in the cytoplasm (Fig. 2E). Thus, *LINC00707* lncRNA, once transcribed, selects the cytoplasm as stable residence.

### **Mechanism of*LINC00707*down-regulation by TGFβ signaling**

**Fig. 3:**
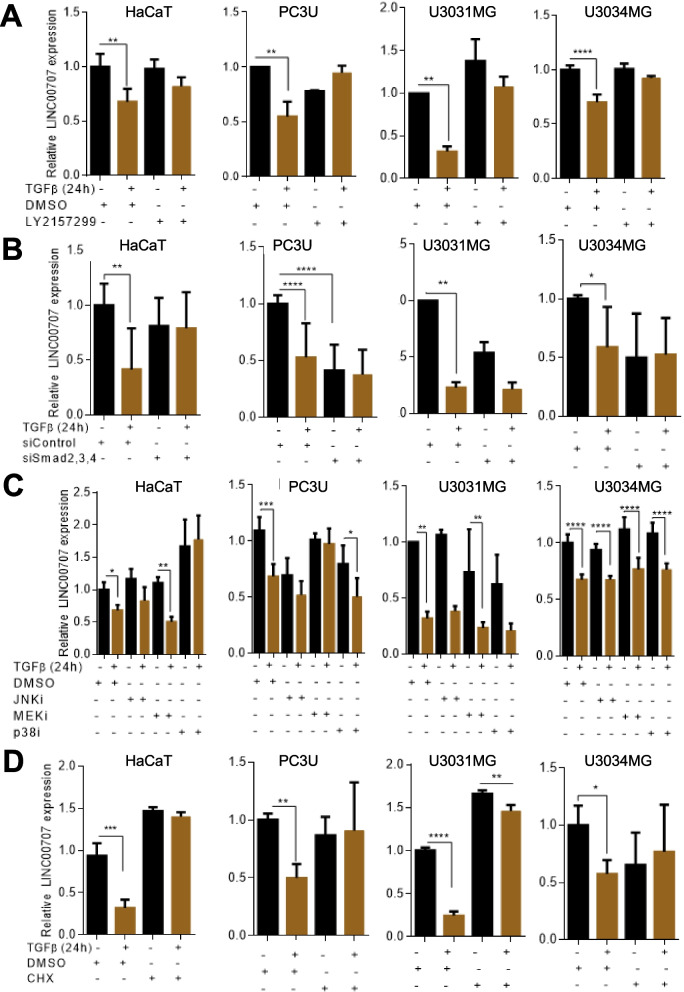
TGFβ-mediated *LINC0707* down-regulation is Smad2/3/4-, MEK- and protein synthesis-dependent. **A-D** RT-qPCR analysis for *LINC00707* in the indicated cell lines after treatment with DMSO (vehicle) or TβRI kinase inhibitor LY2157299 (2.5 nM) (**A**); after transient transfection with siRNAs targeting simultaneously Smad2, Smad3 and Smad4 (**B**); after treatment with DMSO (vehicle) or JNK (JNKi, 10 µM), MEK (MEKi, 0.5 µM) or p38 (p38i, 10 µM) inhibitors (**C**); after treatment with DMSO (vehicle) or cycloheximide (CHX, 40 µg/mL) (D). The cells were also treated or not with TGFβ for 24 h (**A**-**D**). All graphs present data from (*n* = 3) biological repeats, each with three technical repeats, as means with SEM and associated significance as: * *p* < 0.05, ** *p* < 0.01, *** *p* < 0.001, **** *p* < 0.0001. Statistical significance of the variables was assessed by ANOVA test with Bonferroni correction

We then investigated whether inhibition of different TGFβ-induced signaling pathways affected the reduction of *LINC00707* expression in PC3U, HaCaT, U3031MG and U3034MG cells by TGFβ (Fig. 3). The chemical inhibitor of the TβRI kinase, LY2157299, prevented *LINC00707* down-regulation in all cell models, attesting that the kinase activity of TβRI was required (Fig. 3A). Simultaneous silencing of Smad2, Smad3 and Smad4 (Additional file 5, Fig. S3) significantly down-regulated *LINC00707* basal expression, especially in PC3U cells, while further stimulation with TGFβ did not cause any additional effects (Fig. 3B). A chemical inhibitor of the MEK kinase that activates the MAPKs ERK1 and ERK2, weakly reduced basal *LINC00707* expression and eliminated its TGFβ-mediated down-regulation in PC3U cells (Fig. 3C). In HaCaT cells, a c-Jun N-terminal kinase (JNK) inhibitor (JNKi) and a p38 MAPK inhibitor (p38i), completely blocked *LINC00707* down-regulation by TGFβ (Fig. 3C). On the other hand, MEK inhibition did not block *LINC00707* down-regulation, in contrast to the PC3U cell results (Fig. 3C). In U3031MG cells, p38i partially blocked *LINC00707* down-regulation by TGFβ while in U3034MG cells, none of these inhibitors showed a significant effect on *LINC00707* down-regulation by TGFβ, suggesting involvement of different MAPKs in different cell types. Finally, treatment of HaCaT, PC3U, U3031MG and U3034MG cells with the protein synthesis inhibitor cycloheximide blocked the TGFβ-mediated down-regulation of *LINC00707*, indicating requirement of a newly synthesized protein for this down-regulation (Fig. 3D).

We suggest that TGFβ down-regulates *LINC00707* via TβRI, Smad, MAPK signaling and newly synthesized protein(s). Smad and MAPK signaling also modulate basal *LINC00707* expression.

### **TGFβ signaling disengages KLF6 to down-regulate*LINC00707***

**Fig. 4:**
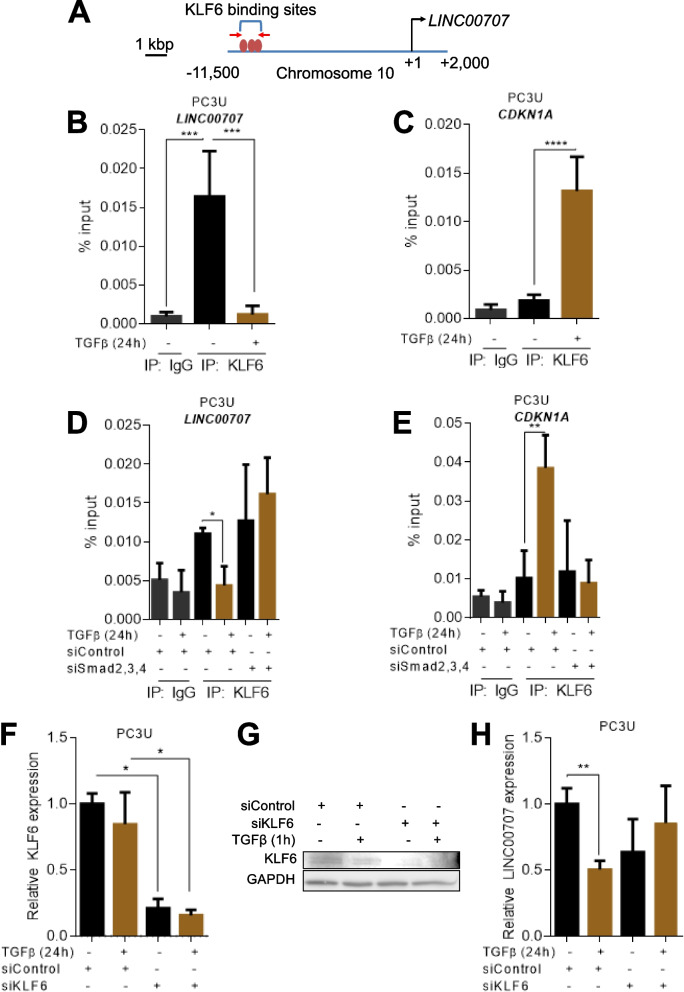
TGFβ signaling displaces KLF6 from the *LINC0707* gene. **A** Schematic representation of the *LINC00707* genomic sequence around its TSS. Predicted KLF6 binding sites are depicted in orange. Red arrows represent the localization of primers for ChIP-qPCR. **B-E** ChIP-qPCR analysis for KLF6 occupancy to the *LINC00707* enhancer in PC3U cells (B); to the *CDKN1A* promoter in PC3U cells (C); to the *LINC00707* enhancer in PC3U cells transiently transfected with siRNAs targeting simultaneously Smad2, 3 ,4 or control siRNA (D); and analysis for KLF6 occupancy to the *CDKN1A* promoter in PC3U cells transiently transfected with siRNAs targeting simultaneously Smad2, 3, 4 or control siRNA (E). Negative control IgG immunoprecipitations are also shown. Values are expressed in % to the input. **F** qRT-PCR for determination of *KLF6* mRNA levels in PC3U cells transiently transfected with siRNA targeting KLF6 or control siRNA. **G** Immunoblot for KLF6 in PC3U cells transiently transfected with control or *KLF6*-targeting siRNA. GAPDH serves as loading control and molecular size markers in kDa are shown. **H** RT-qPCR analysis for *LINC00707* in PC3U cells transiently transfected with siRNA targeting KLF6 or control siRNA. The cells were treated without or with TGFβ for 24 h (B-H). All graphs present data from (*n* = 3) biological replicates, each with three technical repeats, as means with SEM and associated significance as: * *p* < 0.05, ** *p* < 0.01, *** *p* < 0.01, **** *p* < 0.0001. Statistical significance of the variables was assessed by ANOVA test with Bonferroni correction

In order to identify transcriptional mechanisms of *LINC00707* down-regulation by TGFβ, we inspected the *LINC00707* gene sequence, spanning from − 10,000 to + 1,000 bp relative to the transcriptional start site (TSS), in Transfac (https://genexplain.com/transfac/) that predicts transcription factor DNA-binding motifs. Among many predicted motifs, we focused on the Krüppel-like factor-6 (KLF6) whose functions have previously been linked to gene regulation by TGFβ signaling. KLF6 is stabilized by TGFβ signaling, and forms complexes with Smad3 via the zinc-finger transcription factor Sp1 [23], the latter regulating many of the gene responses to TGFβ [24]. KLF6 binding motifs map approximately 8 kbp upstream from the *LINC00707* TSS (Fig. 4A). ChIP assay revealed strong association of KLF6 with the upstream region of the *LINC00707* gene in PC3U cells (Fig. 4B). TGFβ signaling strongly reduced KLF6 binding to the *LINC00707* region (Fig. 4B), suggesting that KLF6 positively regulates expression of *LINC00707* and its removal is necessary for down-regulation of this gene by TGFβ. As a parallel mechanism operating under the identical signaling conditions, we analyzed the *CDKN1A* gene, which is also directly regulated by the Sp1-KLF6 complex [23], and showed strong binding of KLF6 to its promoter, but after TGFβ stimulation (Fig. 4C). We found that Smads are important for maintaining basal *LINC00707* expression (Fig. 3B). KLF6 ChIP-qPCR in PC3U cells after Smad2, 3, 4 triple knock-down showed increased KLF6 binding to the upstream *LINC00707* region; further TGFβ stimulation could not reduce KLF6 binding to the *LINC00707* upstream region (Fig. 4D). Inversely, knock-down of Smad2,3,4 effectively blocked the TGFβ-dependent recruitment of KLF6 to the *CDKN1A* promoter (Fig. 4E). Thus, active Smad-mediated TGFβ signaling is required for KLF6 removal from the *LINC00707* upstream region and subsequent *LINC00707* repression. Accordingly, KLF6 silencing (Fig. 4F, G) reduced basal *LINC00707* expression; under these conditions, *LINC00707* down-regulation by TGFβ was also partially neutralized (Fig. 4H). Thus, TGFβ signaling seems to antagonize the positive action of KLF6 on *LINC00707* expression.

### ***LINC00707*suppresses tumor cell migration and expression of pro-invasive genes**

**Fig. 5:**
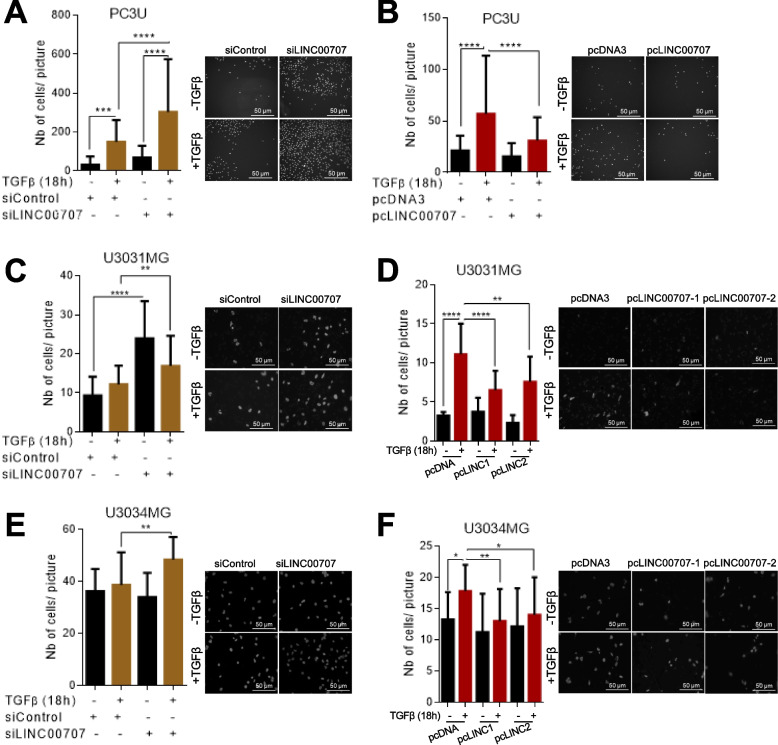
*LINC00707* negatively regulates tumor cell invasion. **A**,** B** Transwell-based collagen invasion assays of PC3U cells transiently transfected with siRNA targeting *LINC00707* or control siRNA (A) or transiently transfected with pc*LINC00707* or control pcDNA3 (B). Cells were treated or not with TGFβ for 18 h during the invasion period. **C-F** Transwell-based laminin invasion assays of U3031MG (C, D) or U3034MG (E, F) cells transiently transfected with siRNA targeting *LINC00707* or control siRNA (C, E) or stably transfected with pc*LINC00707* (pcLINC1, pcLINC2) or control pcDNA3 (D, F). Cells were treated or not with TGFβ for 18 h. The graphs present data from (*n* = 6, PC3U; *n* = 2, U3031MG; *n* = 3, U3034MG) biological replicates, each with three technical replicates, and each replicate representing the sum of 10 random areas of each transwell filter. Statistical significance of the variables was assessed by ANOVA test with Bonferroni correction and is presented as means with SEM and associated significance as: * *p* < 0.05, ** *p* < 0.01, *** *p* < 0.001, **** *p* < 0.0001. Representative photomicrographs are also shown indicating DAPI-positive nuclei. Magnification bars (50 μm). The difference in nuclear size is due to the comparison of different cell types (prostate cancer PC3U cells or brain tumor cells (U3031MG, U3034MG)). The difference in nuclear size between panels of the same cell line is due to the different cell density on each cell culture; dense cultures present nuclei with “smaller” and sparse cultures present nuclei with “larger” circumference as observed under 2D imaging

We examined functional roles of *LINC00707* in the context of TGFβ-regulated tumor biology, and focused on cell invasion since this process is regulated by *LINC00707* in carcinomas and gliomas [25–30]. Silencing endogenous *LINC00707* in PC3U cells (Additional file 5, Fig. S4A) weakly enhanced cell invasion, but strongly enhanced the TGFβ-induced cell invasion (Fig. 5A). Conversely, ectopic *LINC00707* expression (Additional file 5, Fig. S4B) suppressed TGFβ-induced PC3U cell invasion (Fig. 5B). In U3031MG cells, silencing *LINC00707* (Additional file 5, Fig. S4C) enhanced basal invasion by the cells and enhanced TGFβ-induced cell migration (Fig. 5C). We stably transfected U3031MG cells with pc*LINC00707* and selected two clones (pcLINC1 and pcLINC2) overexpressing *LINC00707* (Additional file 5, Fig. S4D). Similar to PC3U cells, ectopic *LINC00707* expression reduced TGFβ-induced cell invasion (Fig. 5D). Silencing of *LINC00707* (Additional file 5, Fig. S4E) and ectopic expression (Additional file 5, Fig. S4F) in U3034MG cells reproduced the results in the other cell types (Fig. 5E, F).

**Fig. 6:**
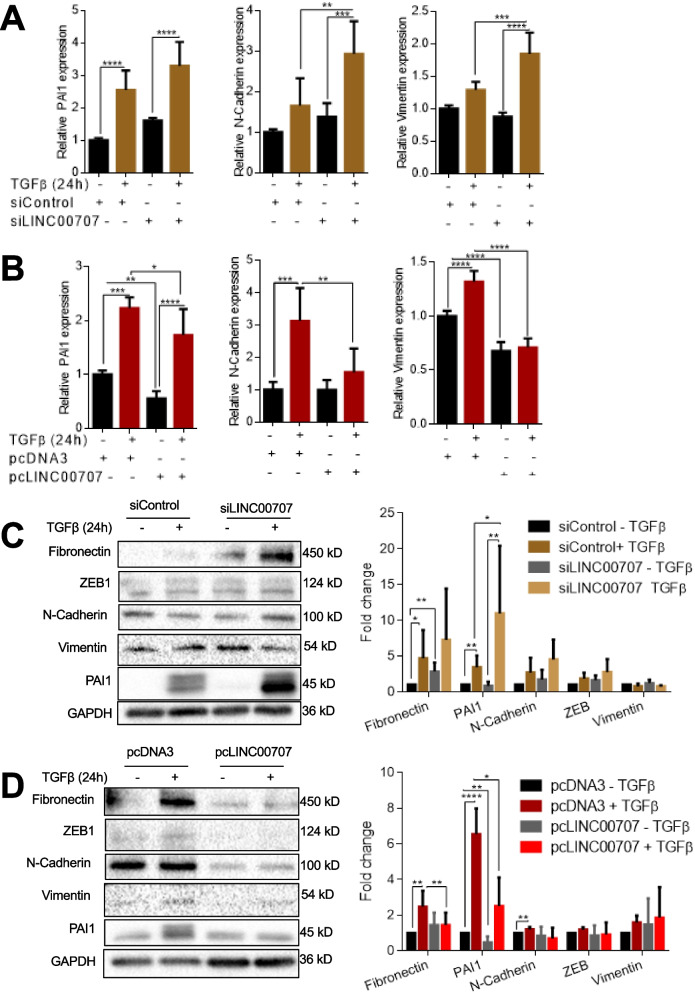
*LINC00707* negatively regulates TGFβ-regulated gene expression. **A**,** B** RT-qPCR analysis for *PAI1*, *N-cadherin* and *vimentin* in PC3U cells transiently transfected with siRNA targeting *LINC00707* or control siRNA (A), or transiently transfected with pc*LINC00707* or control pcDNA3 (B). **C**,** D** Immunoblot and quantification for the indicated proteins (fibronectin, ZEB1, PAI1, N-cadherin) in PC3U cells transiently transfected with siRNA targeting *LINC00707* or control siRNA (C), or in PC3U cells transiently transfected with pc*LINC00707* or control pcDNA3 (D). Cells were treated or not with TGFβ for 24 h (A-D). GAPDH serves as loading control and molecular size markers in kDa are shown. The graphs present data from (*n* = 3, each with three technical repeats for RT-qPCR and *n* = 6 for immunoblots) biological replicates as means with SEM and associated significance as: * *p* < 0.05, ** *p* < 0.01, *** *p* < 0.01, **** *p* < 0.0001, ns: not significant difference. Statistical significance of the variables was assessed by ANOVA test with Bonferroni correction

TGFβ regulates tumor cell invasion via a set of genes of the fibrogenic program [3]. Furthermore, factors that promote carcinoma cell migration and invasion are often implicated in mesenchymal differentiation due to epithelial-mesenchymal transition (EMT), a process potently induced by TGFβ [1]. Silencing *LINC00707* enhanced the basal and TGFβ-induced expression of mRNAs encoding for fibrogenic and EMT factors, including the *plasminogen activator inhibitor 1* (*PAI1*), *N-cadherin* and *vimentin* (Fig. 6A). Moreover, *LINC00707* overexpression suppressed the basal and TGFβ-induced expression of the same mRNAs (Fig. 6B). At the protein level, knock-down and overexpression of *LINC00707* enhanced or suppressed, respectively, the expression of fibronectin, the EMT transcription factor ZEB1, PAI1 and N-cadherin (Fig. 6C, D). These data agree with previous reports from liver, breast and colon carcinoma cells, and demonstrate that *LINC00707* plays a suppressive role on prostate carcinoma cell migration and on pro-invasive and mesenchymal factors, which respond positively to TGFβ signaling.

TGFβ-induced cell invasion is often associated with G1 phase cell cycle arrest, since TGFβ down-regulates the G1 cyclins and up-regulates the cyclin-dependent kinase inhibitors (CKI) p15, p16, p21 and p27 [3]. We investigated PC3U cell proliferation after *LINC00707* knock-down or overexpression (Additional file 5, Fig. S5) by measuring incorporation of Edu into newly synthesized DNA. PC3U cells stimulated with TGFβ exhibited the expected inhibition of cell proliferation; *LINC00707* silencing led to decreased cell proliferation without any further decrease after TGFβ treatment (Additional file 5, Fig. S5A). Consistently, *LINC00707* overexpression led to higher proliferative capacity and no further significant effect of TGFβ (Additional file 5, Fig. S5B). Moreover, immunoblotting showed that the TGFβ-induced increase in CKI p21 (CDKN1A) and decrease of the late G1 phase cyclin E, were further enhanced after *LINC00707* silencing. Conversely, despite TGFβ stimulation, CDKN1A was decreased and cyclin E was increased in PC3U cells overexpressing *LINC00707* (Additional file 5, Fig. S5C).

The above results showed that *LINC00707* modulates the cell invasion capacity of cancer cells via negative regulation of TGFβ-induced target genes, which is associated with a positive effect on cell proliferation. Overall, these data also indicated a general inhibitory effect of *LINC00707* on TGFβ responses.

### ***LINC00707*interacts with Smads and regulates their subcellular localization**

**Fig. 7:**
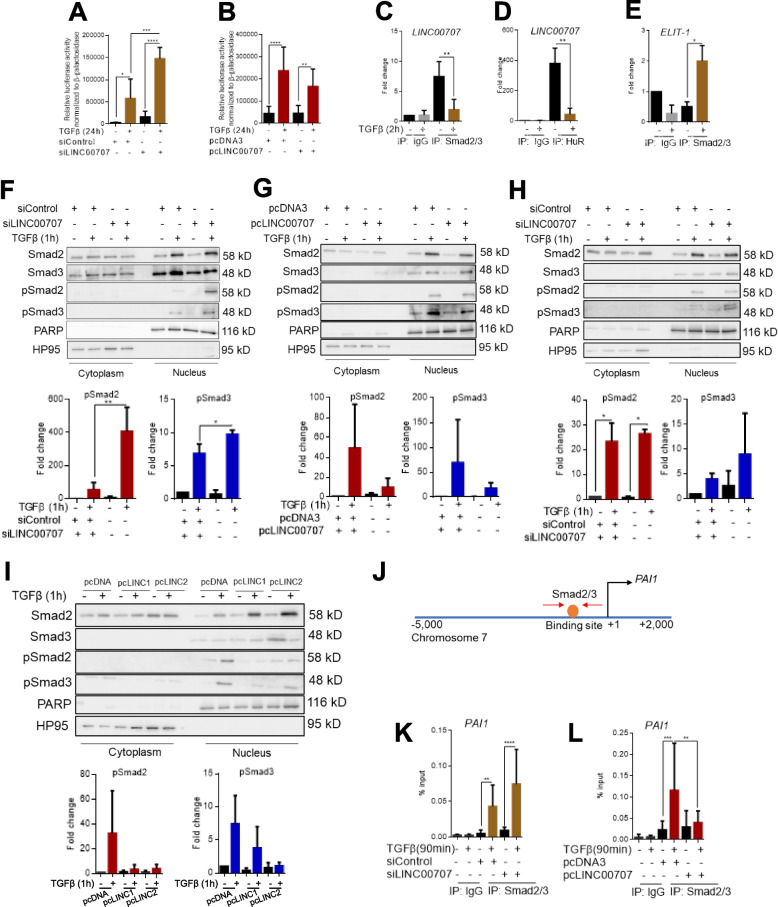
*LINC00707* associates with Smad proteins and negatively regulates TGFβ signaling. **A, B** CAGA_12_-luciferase reporter assay in PC3U cells transiently transfected with siRNA targeting *LINC00707* or control siRNA (A), or transiently transfected with pc*LINC00707* or control pcDNA3 (B). Cells were treated or not with TGFβ for 24 h. **C-E** RIP-qPCR analysis to determine endogenous Smad2/3 association to *LINC00707* (C), endogenous HuR association to *LINC00707* (D), and endogenous Smad2/3 association to *ELIT1* (E) in PC3U cells. Cells were treated without or with TGFβ for 2 h. Negative control IgG immunoprecipitations are shown. Values are expressed in fold-change relative to the negative control (IgG) immunoprecipitations. **F-I** Representative immunoblots of fractions of PC3U (F,G) or U3034MG (H, I) cells for the indicated proteins (Smad2, 3, pSmad2, 3), in cells transiently transfected with siRNA targeting *LINC00707* or control siRNA (F, H) or transiently transfected with pc*LINC00707* or control pcDNA3 (G, I). Cells were treated or not with TGFβ for 1 h. PARP-1 (PARP) and HP95 serve as loading controls of the nuclear and cytoplasmic fractions respectively. Molecular size markers in kDa are shown. Quantification of the pSmad2 and pSmad3 protein bands accompanies the immunoblots. **J** Schematic representation of the *PAI1* promoter. Experimentally verified Smad2/3 binding site is shown in orange. Red arrows represent the localization of primers for ChIP-qPCR. **K, L** ChIP-qPCR analysis for Smad2/3 occupancy to the *PAI1* promoter in PC3U cells transiently transfected with siRNAs targeting *LINC00707* or siRNA control (K), or transiently transfected with pc*LINC00707* or control pcDNA3 (L). Cells were treated without or with TGFβ for 1.5 h. Negative control IgG immunoprecipitations are shown. Values are expressed in % to the input. Graphs presented in panels A-E and K-L show data from (*n* = 3) biological replicates, each in three technical replicates, and panels F-I show data from (*n* = 3) biological replicates as means with SEM and associated significance as: * *p* < 0.05, ** *p* < 0.01, *** *p* < 0.01, **** *p* < 0.0001. Statistical significance of the variables was assessed by ANOVA test with Bonferroni correction

The functional results on cell invasion prompted us to examine whether *LINC00707* directly regulates TGFβ signaling, and possibly via Smads. A synthetic promoter in the CAGA_12_-luciferase reporter, captures TGFβ signaling via Smad3 and Smad4 that bind to the promoter and activate luciferase transcription [31]. Transfecting PC3U cells with the CAGA_12_-luciferase reporter showed that TGFβ robustly activated the reporter, and silencing *LINC00707* further enhanced this activation (Fig. 7A). *LINC00707* overexpression reduced reporter activation by TGFβ (Fig. 7B). Such results could be due to a negative effect of *LINC00707* on TGFβ ligand synthesis and secretion, thus providing an endogenous desensitization to autocrine TGFβ production. The mRNA levels of the ligand TGFβ1 was reduced after transient silencing of *LINC00707* but unchanged after its overexpression (Additional file 5, Fig. S6A-B), while the expression of TGFβ2 was reduced after *LINC00707* knock-down or overexpression (Additional file 5, Fig. S6C-D). ELISA using conditioned media of PC3U cells after *LINC00707* silencing or overexpression, measured about 40 ng/ml of secreted bioactive TGFβ1 and 4 ng/ml of bioactive TGFβ2 in the culture medium, while absence or presence of *LINC00707* did not affect these levels (Additional file 5, Fig. S6E, F).

Since *LINC00707* can associate with proteins [32], we explored the database RNAinter (http://www.rnainter.org/) to identify potential *LINC00707*-binding proteins that could explain its effect on TGFβ signaling. Smad2 and Smad3 appeared as potential interactors of *LINC00707* (Additional file 5, Fig. S7A). Smad2 and Smad3 also appeared in the list of potential transcription factors that bind to the promoter regions of the up-regulated genes upon *LINC00707* silencing in HaCaT cells (analyzed by ChEA2016) (Additional file 5, Fig. S7B). RIP assay using a Smad2/3 antibody detected *LINC00707* bound to Smad2/3 in the absence of TGFβ stimulation in PC3U cells (Fig. 7C). Whereas *LINC00707* strongly bound to Smad2/3 in the absence of TGFβ, when the complex resides in the cytoplasm, this interaction was lost upon TGFβ stimulation for 2 h (Fig. 7C). At this time point, *LINC00707* is only weakly down-regulated by TGFβ in PC3U cells (Fig. 1G), but Smad2 and Smad3 are already phosphorylated and accumulated into the nucleus (see below). As positive controls for the RIP assay, *LINC00707* co-precipitated with the RNA-binding protein HuR, an established interactor of *LINC00707* [32] (Fig. 7D), and Smad3 co-precipitated with ELIT-1 (Fig. 7E), a lncRNA that interacts with Smad3 after activation by TGFβ [33]. As an independent demonstration of the *LINC00707*-Smad3 interaction, we also performed the ChOP assay [22], that is based on an inverse to RIP principle, first *LINC00707* was pulled-down using a tilled series of oligonucleotides, and then associated proteins (Smad3 in this case) were examined by immunoblotting (Additional file 5, Fig. S7C). Endogenous Smad3 protein from PC3U cells associated with endogenous *LINC00707* and stimulation with TGFβ for 2 h reduced the association (Additional file 5, Fig. S7C), which is fully compatible with the RIP data (Fig. 7C). As negative control, oligonucleotides against the *E. coli LacZ* mRNA did not result in detectable Smad3 protein (Additional file 5, Fig. S7C).

Nuclear accumulation of Smads is required to regulate TGFβ-responsive genes. Since *LINC00707* was shown to interact with Smad proteins (Fig. 7C; Additional file 5, Fig. S7C), we assessed whether *LINC00707* affected Smad translocation to the nucleus. We examined the translocation of total and phosphorylated Smad2 and Smad3 to the nucleus by immunoblotting cytoplasmic and nuclear fractions of PC3U cells transiently transfected with siRNA against *LINC00707* (Fig. 7F) or after its overexpression (Fig. 7G), and after treatment or not with TGFβ for 1 h. After *LINC00707* silencing, total Smad2 and Smad3, and more readily pSmad2 and pSmad3 showed increased accumulation in the nucleus relative to the controls with a fold-change of 4 and 1.5 respectively (Fig. 7F). Smad nuclear accumulation was significantly decreased after overexpression of *LINC00707* (5 fold-change; Fig. 7G). A similar but weaker trend for increased nuclear pSmad3 after *LINC00707* knock-down was observed in glioblastoma U3034MG cells (Fig. 7H), whereas its overexpression strongly reduced nuclear pSmad2 and pSmad3 (Fig. 7I).

These findings were corroborated by ChIP assays of Smad2/3 recruitment to the *PAI1* promoter (Fig. 7J), a direct transcriptional target of TGFβ/Smad signaling. Using TGFβ stimulation for 90 min, an optimal time-point for Smad complex accumulation onto chromatin [34], the binding of Smad2/3 induced by TGFβ to the *PAI1* promoter was potentiated upon *LINC00707* silencing in PC3U cells (Fig. 7K). Ectopic *LINC00707* expression had opposite effects, with a significant reduction of Smad2/3 binding to the *PAI1* promoter in the presence of TGFβ stimulation (Fig. 7L). These results show that *LINC00707* modulates the expression of TGFβ-target genes by retaining Smad proteins in the cytoplasm, thus preventing their access to chromatin.

### ***LINC00707*expression negatively correlates with Smad2 activation in tumor tissue**

**Fig. 8:**
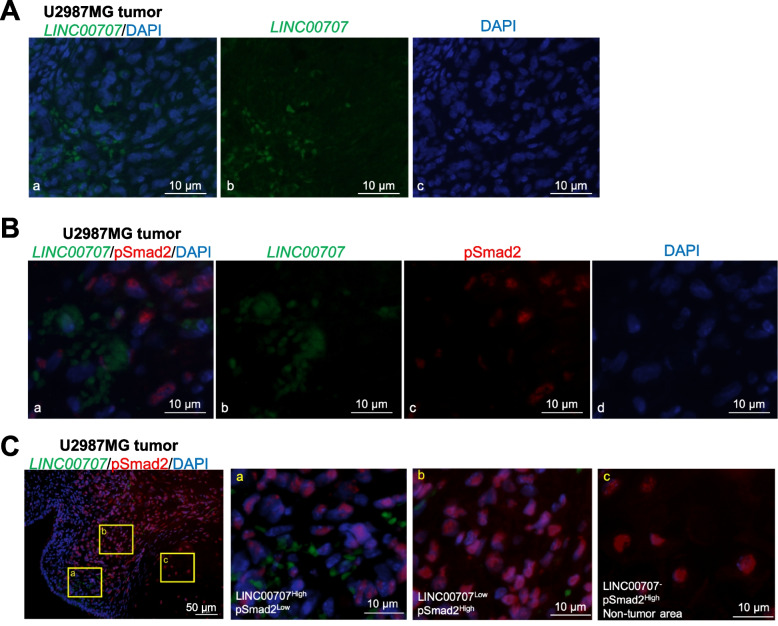
*LINC00707* is expressed in brain tumors and negatively correlates with pSmad2 levels. **A** RNAscope for *LINC00707* expression in a glioblastoma tumor derived from human U2987MG cells orthotopically injected to the mouse brain. Panel **a** shows co-staining for *LINC00707* and DAPI; panels **b** and **c** show the separate channels. **B** RNAscope for *LINC00707* and immunohistochemistry for pSmad2 expression in a glioblastoma tumor derived from human U2987MG cells. Panel **a** shows co-staining for *LINC00707*/pSmad2/DAPI; panels **b** (*LINC00707*), **c** (pSmad2) and **d** (DAPI) show the separate channels. **C** RNAscope for *LINC00707* and immunohistochemistry for pSmad2 expression in a glioblastoma tumor derived from human U2987MG cells. The magnified panel **a** shows a LINC00707^High^/pSmad2^Low^ area, the magnified panel **b** shows a LINC00707^Low^/pSmad2^High^ and the magnified panel **c** shows a LINC00707-negative adjacent mouse tissue area. Scale bars: 50 μm (main panel), 10 μm (magnified panels)

*LINC00707* was detected in tumor tissue obtained from glioblastoma [35] generated after U2987MG-pcDNA3-GFP cells were injected orthotopically in the brain of nude mice (Fig. 8A). Since *LINC00707* modulates Smad translocation to the nucleus, we investigated whether *LINC00707* expression correlated with phosphorylated Smad2 levels using a phospho-Smad2-specific (pSmad2) antibody that generates reliable immunohistochemical detection of endogenous, phosphorylated Smad2 protein (Fig. 8B, C). Simultaneous staining of *LINC00707* by RNAscope and pSmad2 by immunohistochemistry was efficient (Fig. 8B, C). The staining revealed a highly cytoplasmic and rarely nuclear *LINC00707* localization and a varying level of expression among the tumor cells (Fig. 8). Conversely, pSmad2 resided strictly in the nuclei with different intensity, confirming the expected high antibody specificity (Fig. 8B, C). Consistent with our mechanistic results, a negative correlation between *LINC00707* expression and pSmad2 activation was observed in different parts of the tumor (Fig. 8C, insets a and b). According to NCBI, *LINC00707* is only expressed in human tissues, which was confirmed by a negative staining of *LINC00707* in mouse tissue surrounding the tumor (Fig. 8C, inset c). These results show a negative correlation between *LINC00707* expression and pSmad2 activation in human tumors.

### **Novel gene expression profiles regulated by*LINC00707*in the context of TGFβ signaling**

**Fig. 9:**
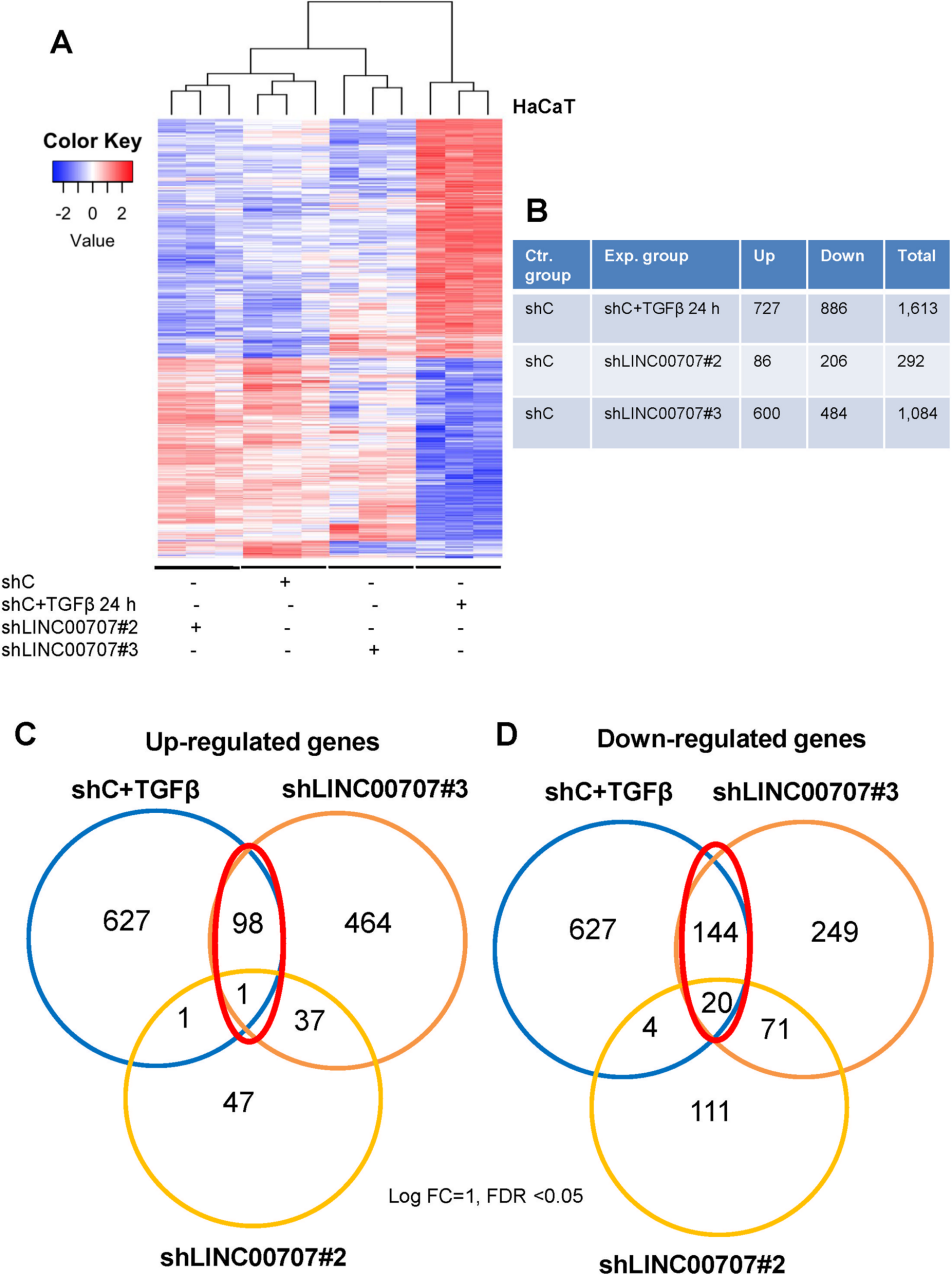
Transcriptomic analysis of human keratinocytes stimulated with TGFβ signaling and after stable silencing of *LINC00707.* **A** Heat-map of the differentially regulated mRNAs in the different experimental conditions as derived by AmpliSeq analysis. A color key indicates expression values in log_2_ scale. **B** Table of the total number of up- or down-regulated genes between the different experimental conditions. **C**,** D** Venn diagrams representing the overlapping up- (C) or down-regulated (D) genes between the different experimental conditions

In order to explore additional genes whose expression was regulated by *LINC00707* and TGFβ, we established HaCaT cell clones with stable *LINC00707* silencing, using two independent shRNAs targeting different regions of *LINC00707* (named as shLINC00707#2 and shLINC00707#3). We verified an appreciable reduction of the *LINC00707* levels in both cell lines (Additional file 5, Fig. S8). We then analyzed the gene expression profiles using AmpliSeq transcriptomics, and compared control HaCaT cells (transfected with a non-targeting shRNA), stimulated or not with TGFβ and the two different *LINC00707*-depleted cell lines (Fig. 9A, B). Depletion of *LINC00707* led to differential gene expression (both enhanced and decreased) (Fig. 9A; Additional files 3 and 4). Silencing of *LINC00707* in shLINC00707#2 cells affected positively expression of 86 genes and negatively of 206 genes, and silencing in shLINC00707#3 cells caused up-regulation of 600 genes and down-regulation of 484 genes (Fig. 9B; Additional files 3 and 4). Stimulation of control (shC) cells with TGFβ for 24 h led to up-regulation of 727 genes, whereas 886 genes were down-regulated (Fig. 9A, B). The significantly higher number of TGFβ-regulated genes compared to the *LINC00707*-regulated genes, may reflect the wider variety of physiological processes affected by TGFβ signaling, compared to the molecular action of *LINC00707*. We were interested in identifying genes commonly regulated by TGFβ and *LINC00707*, as experimental depletion of *LINC00707* recapitulates the repressive effect of TGFβ on *LINC00707* expression. For this reason, we compared the common regulated genes between control cells stimulated with TGFβ, shLINC00707#2 and shLINC00707#3 cells. We observed that only one gene (the transcription factor *zinc finger protein 608* (*ZNF608*)) was commonly up-regulated (Fig. 9C), whereas 20 genes (*APOL6*, *C2CD4A*, *CASP1*, *CFI*, *CLCA2*, *CMPK2*, *CSAG3*, *EIF4E3*, *FAM8A1*, *GBP4*, *IDO1*, *ISLR2*, *FMO3*, *GRIN2A*, *KIT*, *MYO16*, *SERPINB3*, *SYCP2*, *TCN1*, *TSC22D3*) were commonly down-regulated (Fig. 9D) in these three most-stringent experimental conditions. However, analyzing the differentially expressed genes between control and shLINC00707#3, where the silencing efficiency was maximal (Additional file 5, Fig. S8), we enumerated 99 commonly up-regulated and 164 commonly down-regulated genes with TGFβ treatment (Fig. 9C, D; Additional files 3 and 4). Thus, we suggest that *LINC00707* down-regulation by TGFβ signaling results in modulation of specific transcriptional responses.

**Fig. 10:**
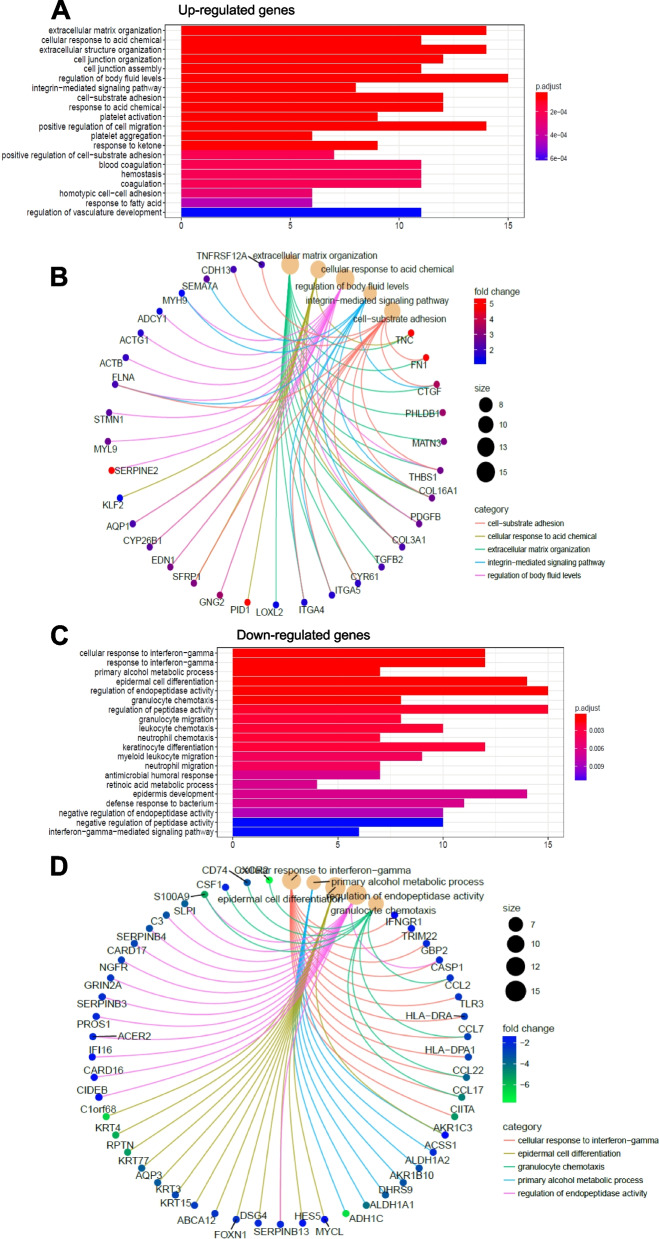
Cell migration- and interferon-γ signaling gene ontologies in human keratinocytes stimulated with TGFβ signaling and after stable silencing of *LINC00707.* **A**,** C** Biological processes of the common up- (A) or down- (C) regulated genes between the shControl + TGFβ, shLINC00707#2 and shLINC00707#3 experimental conditions as derived by GO analysis. Adjusted *p*-value is color-coded. **B, D** GO analysis of the common up- or down-regulated genes between the conditions explained in panels A, C, with representative examples of genes. Fold-change in expression is color-coded and circle diameters indicate normalized number (count) of gene members per category

Gene ontology analysis of the commonly up-regulated (99) and down-regulated (164) genes between control cells stimulated with TGFβ, and shLINC00707#3 cells (Fig. 10), showed up-regulated genes involved in processes of extracellular matrix organization, cell junction assembly and regulation of cell migration (Fig. 10A), such as *FN1*, *COL3A1*, *SERPINE2* and *CDH13* (Fig. 10B). These data agree with the impact of *LINC00707* on TGFβ-mediated pro-migratory and mesenchymal differentiation responses (Figs. 5 and 6). Interestingly, the top down-regulated genes are related to the cellular response to interferon-gamma (IFN-γ) and the IFN-γ-mediated signaling pathway (Fig. 10C, D), suggesting a possible role of *LINC00707* on immune cell activation. Thus, *LINC00707* regulates the expression of several pro-migratory and adhesion-related genes, yet it does not capture the complete repertoire of TGFβ-mediated gene expression profile.

## Discussion

By combining transcriptomic data from normal and tumor cells that potently respond to TGFβ, we identified that *LINC00707* is a TGFβ-target gene (Fig. 1). The regulation of *LINC00707* expression by TGFβ is not restricted to a particular cell type, as *LINC00707* is down-regulated in a large variety of tumor and normal cells types (Fig. 1F). *LINC00707* is located mainly in the cytoplasm of diverse cell types (Fig. 2). We provide new evidence about the regulation of *LINC00707* expression by growth factor pathways.

Many lncRNAs regulate transcriptionally or post-transcriptionally the expression of genes, which are located at distal places relative to the lncRNA genomic location, via a *trans* mechanism [36]. On the other hand, cytoplasmic lncRNAs function as molecular sponges for micro-RNAs (miRNAs), by base-pairing with complementary RNA sequences, thus stabilizing specific mRNAs [37]. Previous reports established that *LINC00707* is highly expressed in lung and hepatocellular carcinomas, correlates with faster cell proliferation and migration and with increased tumor size, metastasis and poor prognosis of these tumors [25, 26]. Micro-RNA sponging has been proposed as a function of *LINC00707*; *miR-206* base-pairs to *LINC0070*7 and is thus inactivated in colorectal and breast cancer cells [27–29]. Since *miR-206* is multifunctional, mechanisms by which *LINC00707* affects cancer cell functions involves various *miR-206*-target mRNAs; NOTCH3 controls cell proliferation, and formin-like 2 regulates cell migration [28, 29]. In gliomas, *LINC00707* promotes cell migration and proliferation, possibly by interfering with the function of *miR-613* [30]. In additional cancer cell types *LINC00707* sponges *miR-30c* [38], *miR-145* [39], *miR-270* [40] and *miR-485-5p* [41].

In addition, lncRNAs may bind proteins, such as transcription factors or cytoplasmic proteins [42]. *LINC00707* can associate with the house-keeping RNA-binding protein HuR in gastric cancer cells (confirmed also here in prostate adenocarcinoma cells, Fig. 7D), which leads to enhanced stabilization of oncogenic mRNAs [32]. The present study shows that *LINC00707* also binds to Smad proteins in the cytoplasm, where it resides, and thus retains Smads in the cytoplasm, limiting the activation of TGFβ signaling (Fig. 7; Additional file 5, Fig. S7C). We therefore suggest that the TGFβ-induced down-regulation of *LINC00707* releases cytoplasmic Smad proteins and facilitates their accumulation in the nucleus and therefore their binding to specific TGFβ-target genes, such as *PAI1* (Fig. 7). In tumor tissue, we demonstrated a negative correlation between expression of cytoplasmic *LINC00707* and phosphorylated Smad2 (Fig. 8). This suggests that the *LINC00707*-Smad mechanism is relevant to human cancer biology in vivo; it remains open to examine whether specific cell types within a tissue present the *LINC00707*-Smad mechanism.

TGFβ signaling is a process that requires fine-tuning [3]. The engagement of lncRNAs in this process via their interaction with Smads or via alternative mechanisms leads to enhanced or diminished activation of TGFβ signaling [8–10]. Some of these lncRNAs interact with Smads and form positive or negative feedback loops. For example, *ELIT-1* is induced by TGFβ and interacts with Smad3 in order to facilitate Smad recruitment to target genes, thereby promoting the expression of EMT-related genes [33]. Conversely, the *lncRNA-TSI* binds to Smad3 and blocks its phosphorylation by TβRI, resulting in a negative feedback loop [43]. *NORAD* is a cytoplasmic lncRNA that associates with importin-β1 [44], the carrier of Smad3 to the nucleus [45], and thus facilitates nuclear accumulation of Smad3 and positively contributes to TGFβ signaling. In this context, the identification of *LINC00707* as a regulator of Smad translocation to the nucleus reveals a new mechanism of regulation of TGFβ signaling, whereby *LINC00707* interacts with the Smads in the cytoplasm, acting as a brake for their translocation to the nucleus. TGFβ both enhances the phosphorylation of the Smad complex and induces down-regulation of *LINC00707* to prolong its signaling. Thus, *LINC00707* is implicated in multiple facets of regulation during TGFβ signaling.

The mechanism of down-regulation of *LINC00707* by TGFβ involves multiple components; the Smad proteins, MAPKs and the transcription factor KLF6 (Figs. 3 and 4). This is compatible with the mechanism of regulation of many other target genes of the TGFβ pathway [3]. In particular, genes whose expression is down-regulated by TGFβ are often indirectly regulated and require new protein synthesis [46], as attested also here for *LINC00707* after cycloheximide blocking experiments (Fig. 3D). The cycloheximide experiments suggest a role of an as yet unidentified newly synthesized protein required for *LINC00707* down-regulation. Furthermore, Smads and MAPKs contribute to basal *LINC00707* expression. Our analysis of signaling pathways contributing to regulation of *LINC00707* expression (Fig. 3) have also indicated subtle differences between normal or cancer cell models of different tissue origin, as is the case of the contribution of JNK, MEK and p38 MAPK in HaCaT, PC3U and GBM cells (Fig. 3C). Such differences are frequently reported in the literature and often reflect tissue of origin or cancer type differences, including the contribution of the individual patient donor. The data also suggest that combined Smad and MAPK signals may delocalize the positive regulator KLF6 from the *LINC00707* promoter, causing transcriptional repression (Figs. 3 and 4). This model is compatible with the established interaction between KLF6 and Smads and the phosphorylation of KLF6 by MAPKs [23, 34]. Whether different tissue or tumor types engage different MAPK family members to regulate the same transcription factor (KLF6) remains to be examined.

*LINC00707* has been shown to act as an oncogene, which is up-regulated in lung adenocarcinoma and hepatocarcinoma tissues and its overexpression correlates with higher cell proliferation, migration, tumor size, metastasis and poor prognosis [25, 26]. Cancer cell migration assays and mesenchymal marker analysis demonstrated a negative role of *LINC00707* on TGFβ-mediated cell migration and mesenchymal protein expression in prostate cancer and glioblastoma cells (Figs. 5 and 6). This agrees with our transcriptomic analysis performed in HaCaT cells, which showed that silencing *LINC00707* resulted in up-regulation of genes encoding extracellular matrix proteins (*FN1*, *TNC*, *SERPINE2*, LOXL2), fibrogenic and pro-migratory growth factors (*TGFB2*, *PDGFB*, *CTGF*), and integrins (*ITGA2*, *ITGA5*) (Figs. 9 and 10). The negative impact of *LINC00707* on TGFβ-mediated cell migration and mesenchymal differentiation (Figs. 5 and 6), and the positive effects of *LINC00707* on migration in different cancer cell models [25, 26, 28–30, 32, 38], suggest that the mechanism by which *LINC00707* regulates cell migration is modified when cells respond to TGFβ signaling.

## Conclusions

We describe a new mechanism of transcriptional down-regulation of *LINC00707* by TGFβ signaling, as well as a new mechanism of regulation of TGFβ/Smad signaling by *LINC00707.* This adds *LINC00707* to the network of newly identified lncRNAs that participate in positive or negative feedback loops of TGFβ signaling [8–10]. One explanation for the TGFβ-mediated *LINC00707* down-regulation could be the facilitation of maximal signaling activity by TGFβ, a process relevant to sustained TGFβ signaling operating in human tumors [35, 47, 48].

### Supplementary Information

**Additional file 1.**

**Additional file 2.**

**Additional file 3.**

**Additional file 4.**

**Additional file 5.**

## Data Availability

We have previously published all primary microarray [13], AmpliSeq and RNA-sequencing [19] data and were also deposited to ArrayExpress with accession numbers E-MTAB-9076 and E-MTAB-12,980. Further details or additional materials are available upon reasonable request.

## Abbreviations

ChIP: Chromatin immunoprecipitation
ChOP: Chromatin oligo-affinity precipitation
CKI: Cyclin-dependent kinase inhibitor
EMT: Epithelial-mesenchymal transition
JNK: c-Jun N-terminal kinase
KLF6: Krüppel-like factor 6
lncRNA: Long non-coding RNA
MAPK: Mitogen-activated protein kinase
miRNA: Micro-RNA
PAI1: Plasminogen activator inhibitor 1
RIP: RNA immunoprecipitation
shRNA: Short-hairpin RNA
TGFβ: Transforming growth factor β
TSS: Transcription start site
