# Supplementary material for: The long non-coding RNA LINC00707 interacts with Smad proteins to regulate TGFβ signaling and cancer cell invasion
Source: Cell Commun Signal. 2023 Oct 2;21:271. doi: 10.1186/s12964-023-01273-3 (PMC10544626; doi:10.1186/s12964-023-01273-3)
Supplement: Supplementary file 2 — Additional file 1. [file 12964_2023_1273_MOESM1_ESM.pdf]

The long non-coding RNA *LINC00707* interacts with Smad proteins to regulate TGFβ signaling and cancer cell invasion

Caroline Gélabert, Panagiotis Papoutsoglou, Irene Cancela Golan, Eric Ahlström, Adam Ameur, Carl-Henrik Heldin, Laia Caja, and Aristidis Moustakas

Additional Information

Additional file 1.

Table S1: siRNAs and plasmids used for transfections.

siControl	*ON-TARGETplus Non-targeting Pool. Dharmacon Inc./GE Healthcare/Horizon Group, Lafayette, CO, USA.* *Ref: D-001810-10-20*
siSmad2	*ON-TARGETplus Human Smad2 siRNA SMARTpool. Dharmacon Inc./GE Healthcare/Horizon Group, Lafayette, CO, USA*. Ref: L-003561-00-0005
siSmad3	*ON-TARGETplus Human Smad3 siRNA SMARTpool. Dharmacon Inc./GE Healthcare/Horizon Group, Lafayette, CO, USA*. Ref: L-020067-00-0005
siSmad4	*ON-TARGETplus Human Smad4 siRNA SMARTpool. Dharmacon Inc./GE Healthcare/Horizon Group, Lafayette, CO, USA*. Ref: L-003902-00-0005
siLINC00707	*Lincode Human LINC00707 siRNA SMARTpool. Dharmacon Inc./GE Healthcare/Horizon Group, Lafayette, CO, USA*. Ref: R-188521-00-0005
pcDNA3	*Obtained from our lab stock and as previously described* {Papoutsoglou, 2019 #3354}.
pcDNA3-LINC00707	*Constructed in-house as described here*.
siKLF6	*ON-TARGETplus Human KLF6 siRNA SMARTpool. Dharmacon Inc./GE Healthcare/Horizon Group, Lafayette, CO, USA*. Ref: L-021441-00-0005
