# Supplementary material for: The long non-coding RNA LINC00707 interacts with Smad proteins to regulate TGFβ signaling and cancer cell invasion
Source: Cell Commun Signal. 2023 Oct 2;21:271. doi: 10.1186/s12964-023-01273-3 (PMC10544626; doi:10.1186/s12964-023-01273-3)
Supplement: Supplementary file 3 — Additional file 2. [file 12964_2023_1273_MOESM2_ESM.pdf]

Additional file 2.

Table S2: Oligonucleotides used for RT-qPCR, RIP, ChOP and ChIP.

LINC00707 Set1	Eurofins Sweden, Uppsala, Sweden; H455, RT-qPCR, RIP
Fw:	CCT-TCG-GCC-CAT-TTC-TCA-CT
Rev:	ATC-ACG-GTG-GCA-GTA-TCG-TG
LINC00707 Set2	Eurofins Sweden, Uppsala, Sweden, RT-qPCR
Fw:	CCT-TCG-GCC-CAT-TTC-TCA-CT
Rev:	ATC-ACG-GTG-GCA-GTA-TCG-TG
PAI-1	Eurofins Sweden, Uppsala, Sweden; H789, RT-qPCR
Fw:	GAG-ACA-GGC-AGC-TCG-GAT-TC
Rev:	GGC-CTC-CCA-AAG-TGC-AAT-AC
KLF-6	Eurofins Sweden, Uppsala, Sweden; H737, RT-qPCR
Fw:	CAA-GGG-AAA-TGG-CGA-TGC-CT
Rev:	GGC-TTT-TCT-CCT-GTG-TGC-GT
Smad2 Set1	Eurofins Sweden, Uppsala, Sweden, RT-qPCR
Fw:	TGC-CTT-CGG-TAT-TCT-GCT-CCC-CA
Rev:	TGG-CTG-GCA-CCC-TGC-AAC-AG
Smad2 Set2	Eurofins Sweden, Uppsala, Sweden, RT-qPCR
Fw:	TGG-CTG-GCA-CCC-TGC-AAC-AG
Rev:	TGC-CTT-CGG-TAT-TCT-GCT-CCC-CA
Smad3	Eurofins Sweden, Uppsala, Sweden, RT-qPCR
Fw:	GCA-ATA-TTC-CAG-AGA-CCC-CAC-C
Rev:	TAG-GTT-TGG-AGA-ACC-TGC-GTC-C
Smad4	Eurofins Sweden, Uppsala, Sweden, RT-qPCR
Fw:	CAT-CCT-GGA-CAT-TAC-TGG-CCA
Rev:	CCT-ACC-TGA-ACG-TCC-ATT-TCA-A
N-Cadherin	Eurofins Sweden, Uppsala, Sweden, RT-qPCR
