# Supplementary material for: The long non-coding RNA LINC00707 interacts with Smad proteins to regulate TGFβ signaling and cancer cell invasion
Source: Cell Commun Signal. 2023 Oct 2;21:271. doi: 10.1186/s12964-023-01273-3 (PMC10544626; doi:10.1186/s12964-023-01273-3)
Supplement: Supplementary file 6 — Additional file 5. [file 12964_2023_1273_MOESM5_ESM.pdf]

Additional File 5.

Additional figure titles and legends

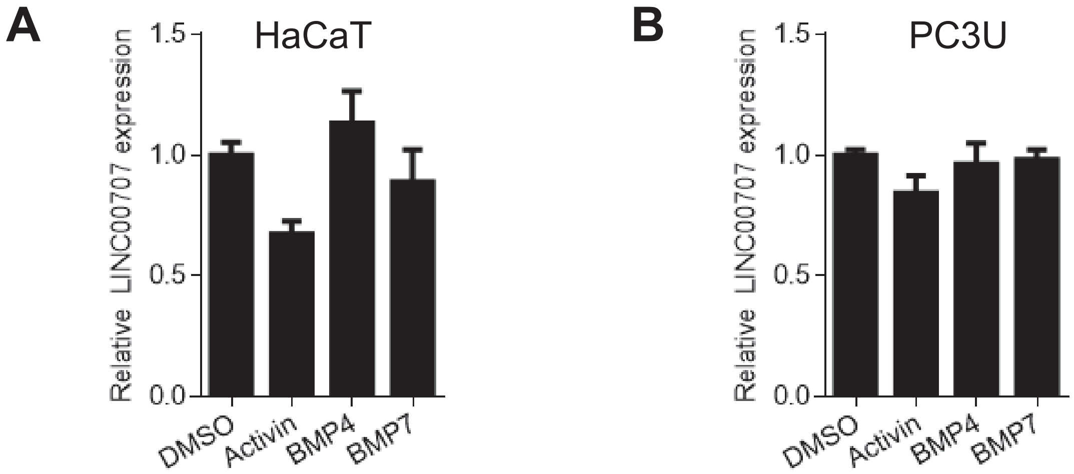

Supplemental Figure 1 related to Main Figure 1

Fig. S1 related to Fig. 1. LINC00707 is specifically regulated by TGFβ. (**A, B**) Real-time RT-PCR for determination of *LINC00707* mRNA levels in HaCaT (A) and PC3U (B) cells treated with activin-A (10 ng/mL), BMP4 (30 ng/mL) or BMP7 (30 ng/mL) for 24 h. Graphs present data from (n=3) biological replicates, each with three technical repeats, as means with SEM and associated significance as: * $p<0.05$, ** $p<0.01$, *** $p<0.01$, **** $p<0.0001$. Statistical significance of the variables was assessed by ANOVA test with Bonferroni correction.

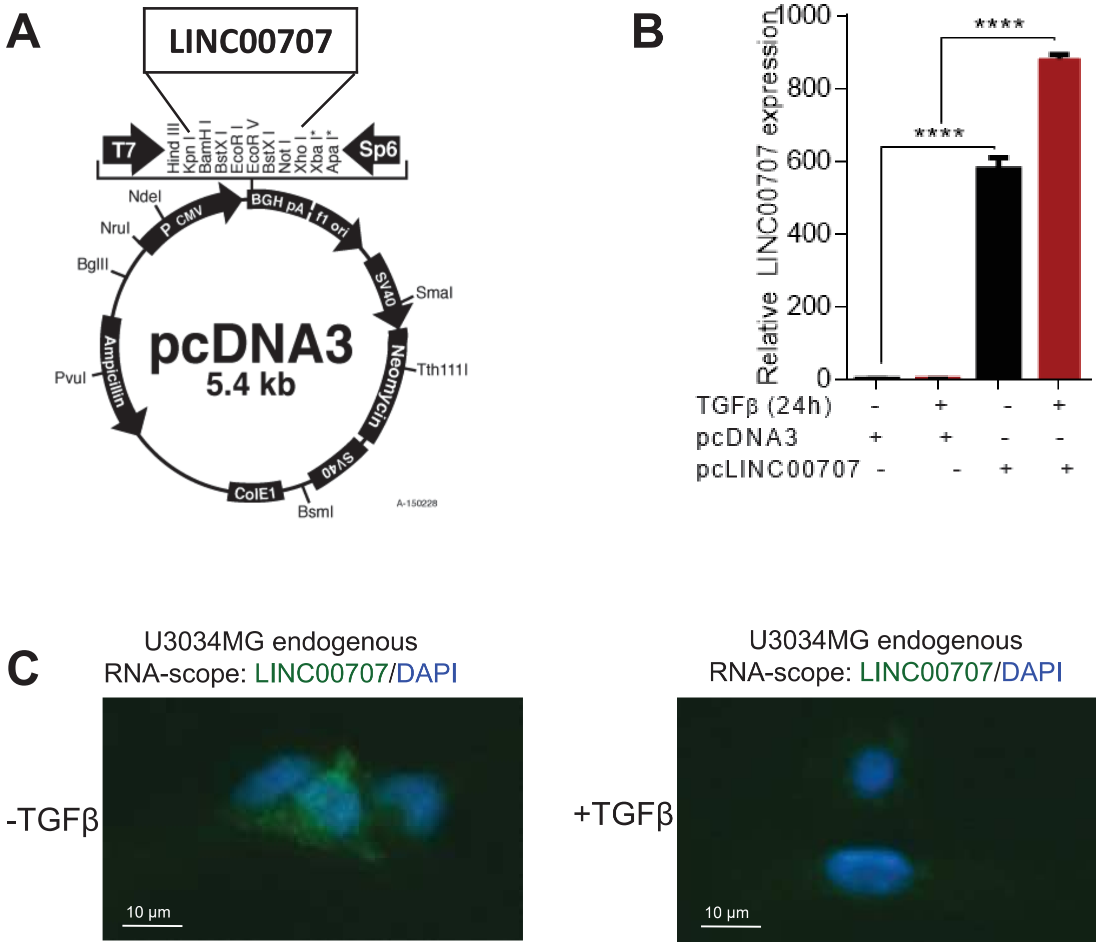

Supplemental Figure 2 related to Main Figure 2

Fig. S2 related to Fig. 2. Efficiency of *LINC00707* overexpression in PC3U cells and regulation by TGFβ in U3034MG. (**A**) Schematic representation of the recombinant pcDNA3-LINC00707 expression vector. (**B**) Real-time RT-PCR for determination of *LINC00707* mRNA levels in PC3U cells transiently transfected with siRNA targeting *LINC00707* or control siRNA and treated with TGFβ for 24 h or not. The graph presents data from one biological replicate with three technical repeats as means with SEM and associated significance as: * p<0.05, ** p<0.01, *** p<0.01, **** p<0.0001. Statistical significance of the variables was assessed by ANOVA test with Bonferroni correction. (**C**) RNAscope for endogenous *LINC00707* in U3034MG cells treated or not with TGFβ for 24 h. Scale bar: 10 μm.

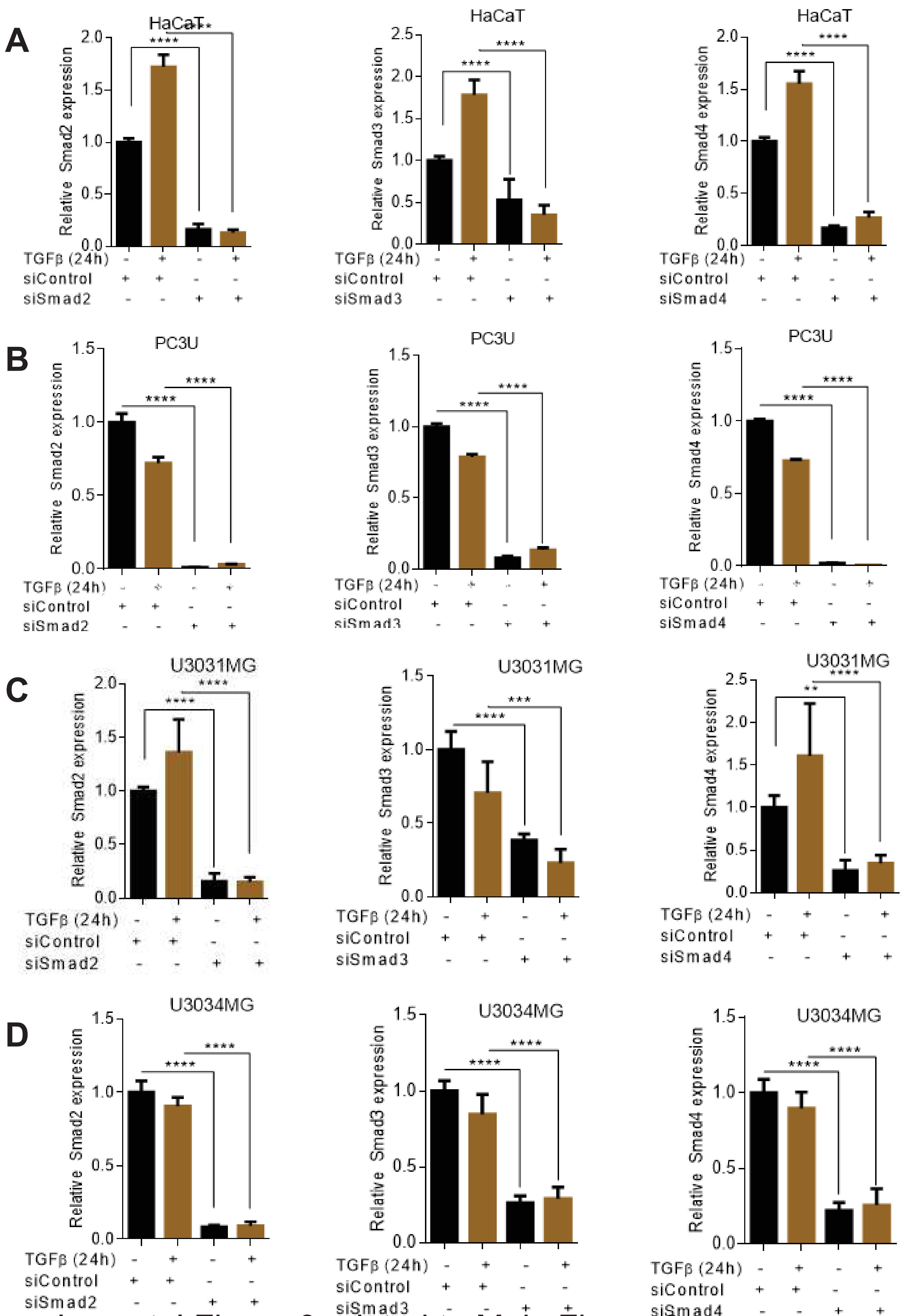

Supplemental Figure 3 related to Main Figure 3

Fig. S3 related to Fig. 3. Efficiency of Smad silencing in PC3U cells. (**A-D**) Real-time RT-PCR for determination of *Smad2*, *Smad3* or *Smad4* mRNA levels in HaCaT (A), PC3U (B), U3031MG (C) and U3034MG (D) cells transiently transfected with siRNAs targeting Smad2, Smad3, Smad4 or control siRNA and treated with TGFβ for 24 h or untreated. All graphs present data from (n=2) biological replicates, each with three technical repeats, as means with SEM and associated significance as: * $p<0.05$, ** $p<0.01$, *** $p<0.01$, **** $p<0.0001$. Statistical significance of the variables was assessed by ANOVA test with Bonferroni correction.

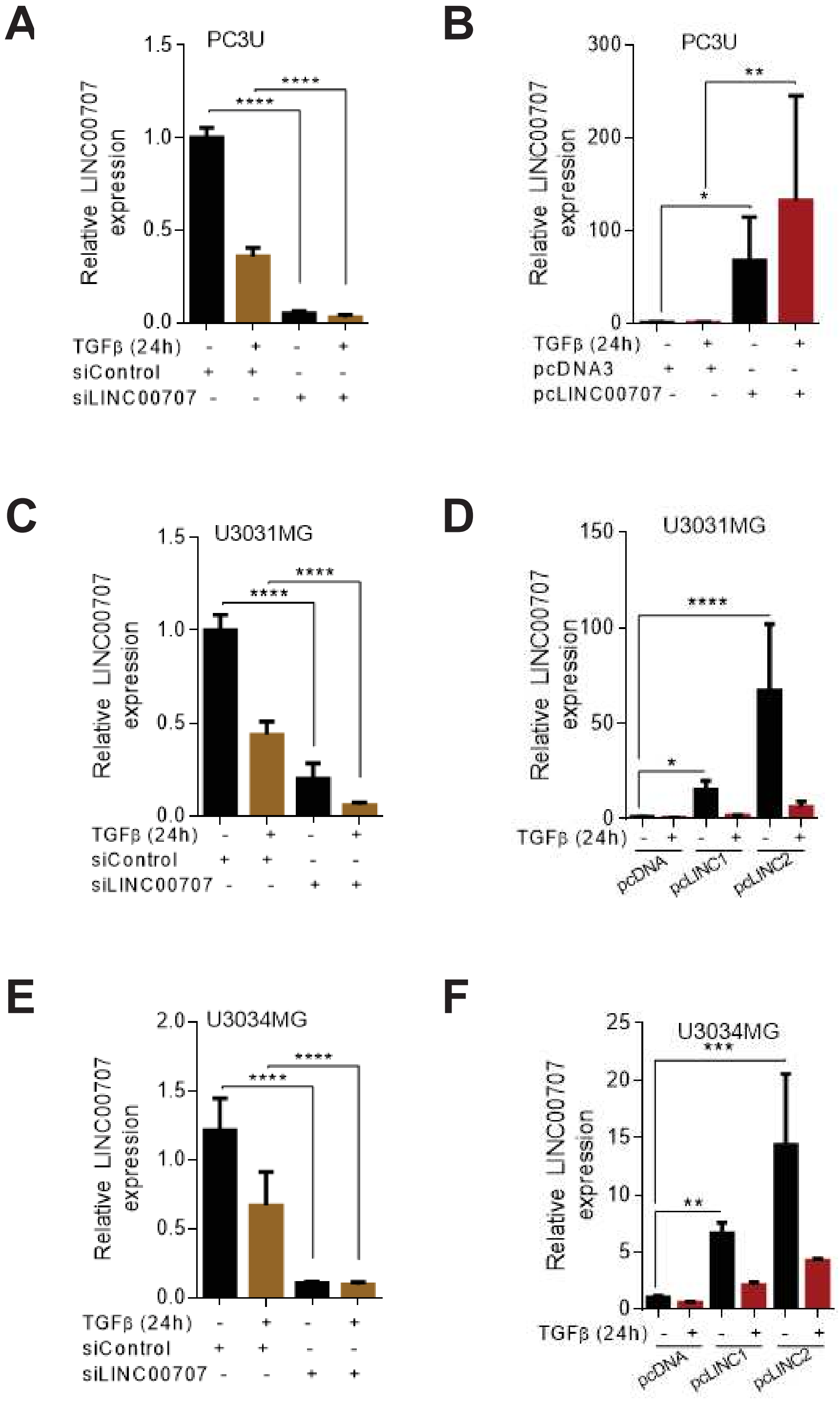

Supplemental Figure 4 related to Main Figure 5

Fig. S4 related to Fig. 5. Efficiency of silencing or overexpression of *LINC00707* in PC3U, U3031MG and U3034MG cells. (**A-F**) Real-time RT-PCR for determination of *LINC00707* mRNA levels in PC3U (A, B), U3031MG (C, D) and U3034MG (E, F) cells transiently transfected with siRNA targeting *LINC00707* or control siRNA (A, C, E), or transiently overexpressing pcDNA3-*LINC00707* or control pcDNA3 (B, D, F). Cells were treated with TGFβ or not for 24 h. All graphs present data from triplicate assays as means with SEM. All graphs present data from (n=2) biological replicates, each with three technical repeats, as means with SEM and associated significance as: * $p<0.05$, ** $p<0.01$, *** $p<0.01$, **** $p<0.0001$. Statistical significance of the variables was assessed by ANOVA test with Bonferroni correction.

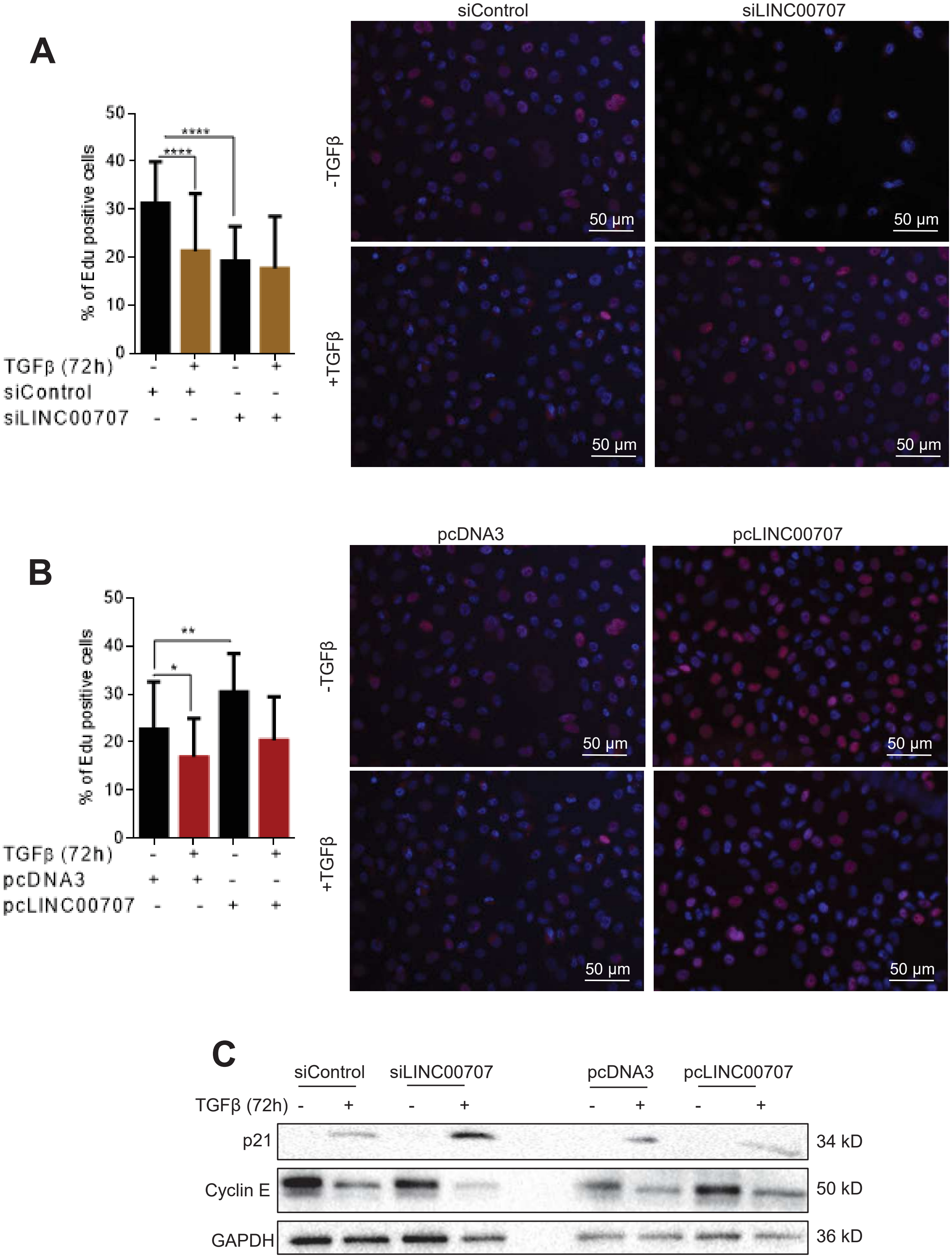

Supplemental Figure 5 related to Main Figure 6

Fig. S5 related to Fig. 6. *LINC00707* positively regulates cell proliferation in PC3U cells. (**A**) Percentage of Edu-positive PC3U cells transiently transfected with siRNA targeting *LINC00707* or control siRNA, and treated with TGFβ for 72 h or untreated. (**B**) Percentage of Edu-positive PC3U cells transiently transfected with pcDNA3-*LINC00707* or control pcDNA3 and treated or not with TGFβ for 72 h. (**C**) Immunoblot for the indicated proteins (CDKN1A, cyclin E) in PC3U cells transiently transfected with pcDNA3-*LINC00707* or control pcDNA3, or, control or *LINC00707*-targeting siRNAs and treated or not with TGFβ for 72 h. GAPDH serves as loading control and molecular size markers in kDa are shown. All graphs present data from (n=3) biological replicates, each with three technical repeats, as means with SEM and associated significance as: * $p<0.05$, ** $p<0.01$, *** $p<0.01$, **** $p<0.0001$. Statistical significance of the variables was assessed by ANOVA test with Bonferroni correction.

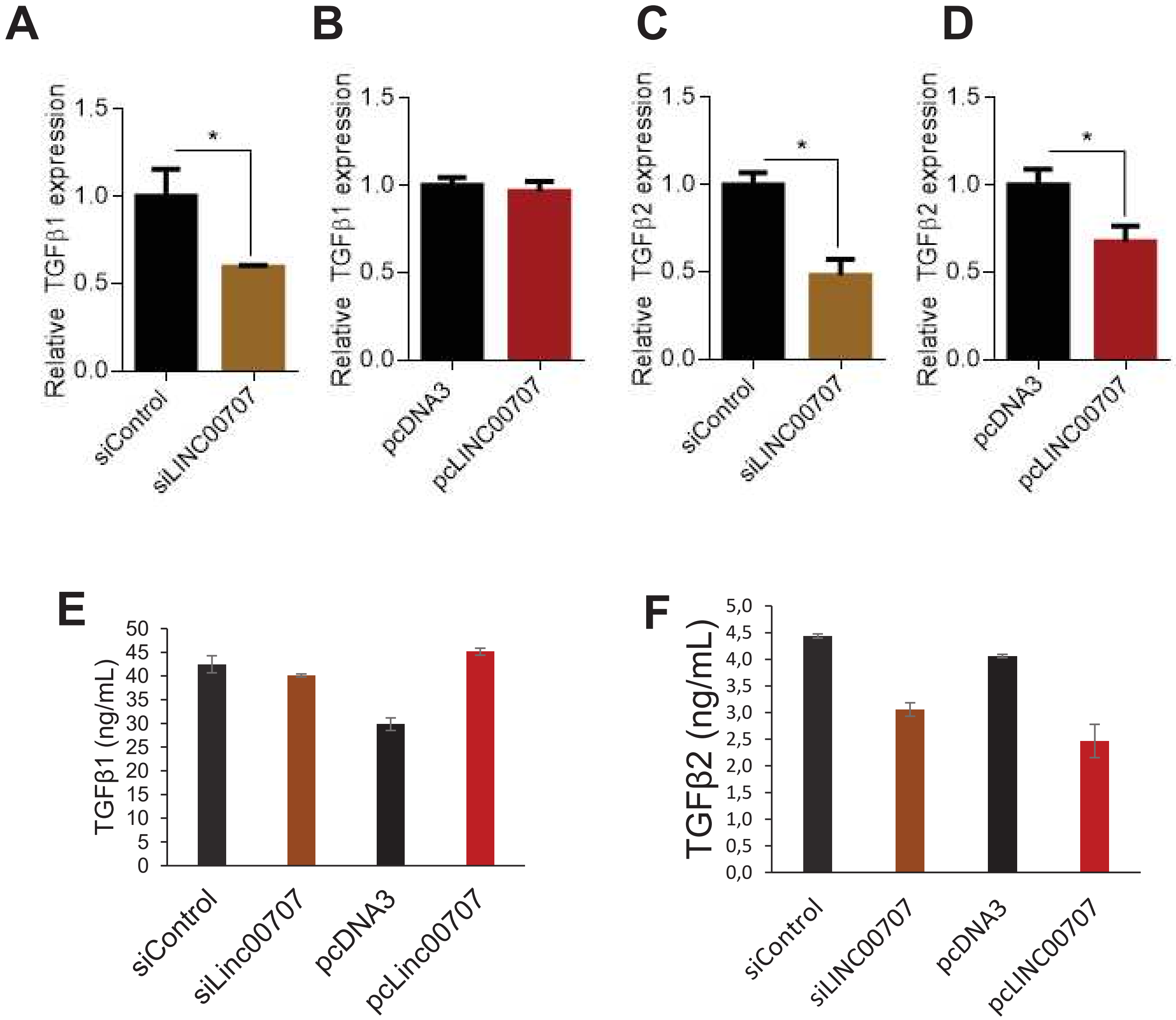

Supplemental Figure 6 related to Main Figure 7

Fig. S6 related to Fig. 7. *LINC00707* does not affect the TGFβ secretion in PC3U cells. (**A, B**) Real-time RT-PCR for determination of *TGFβ1* mRNA levels in PC3U cells transiently transfected with siRNA targeting *LINC00707* or control siRNA (A), or transiently overexpressing pcDNA3-*LINC00707* or control pcDNA3 (B). (**C, D**) Real-time RT-PCR for determination of *TGFβ2* mRNA levels in PC3U cells transiently transfected with siRNA targeting *LINC00707* or control siRNA (C), or transiently overexpressing pcDNA3-*LINC00707* or control pcDNA3 (D). All graphs present data from (n=3) biological replicates, each with three technical repeats, as means with SEM and associated significance as: * $p<0.05$, ** $p<0.01$, *** $p<0.01$, **** $p<0.0001$. Statistical significance of the variables was assessed by unpaired Student's t-test. (**E, F**) Mature human TGFβ1 (E) and TGFβ2 (F) protein levels in conditioned media of PC3U cells transiently transfected with siRNA targeting *LINC00707* or control siRNA, and in PC3U cells transiently transfected with pcDNA3-*LINC00707* or control pcDNA3, determined by ELISAs. No statistical analysis was performed due to the unique biological repeat in three technical repeats and the absence of detectable change in protein levels.

A

Interactor1	Interactor2	Score.
LINC00707	**CTCF**	0.6119
LINC00707	**FOXA1**	0.4757
LINC00707	**SOX2**	0.4442
LINC00707	**ESR1**	0.3961
LINC00707	**TP53**	0.3908
LINC00707	**GATA4**	0.3494
LINC00707	**FOXM1**	0.3383
LINC00707	**EP300**	0.2566
LINC00707	**EZH2**	0.253
LINC00707	**PPARG**	0.2514
LINC00707	**SALL4**	0.25
LINC00707	**SMC3**	0.2469
LINC00707	**SMAD2**	0.2396
LINC00707	**BRD4**	0.2356
LINC00707	**CEBPB**	0.2259
LINC00707	**JUN**	0.2258
LINC00707	**JUND**	0.2219
LINC00707	**ARNT**	0.212
LINC00707	**HIF1A**	0.212
LINC00707	**SMAD3**	0.2117
LINC00707	**STAT3**	0.2041

B

ESR2
RNF2
EZH2
SUZ12
ESR1
UBF1/2
MTF2
SMAD2
SMAD3
GATA1
TCF7
KDM2B
GATA4
RACK7
JARID2
SMAD4
ATF3
CEBPD
SOX2
RELA

C

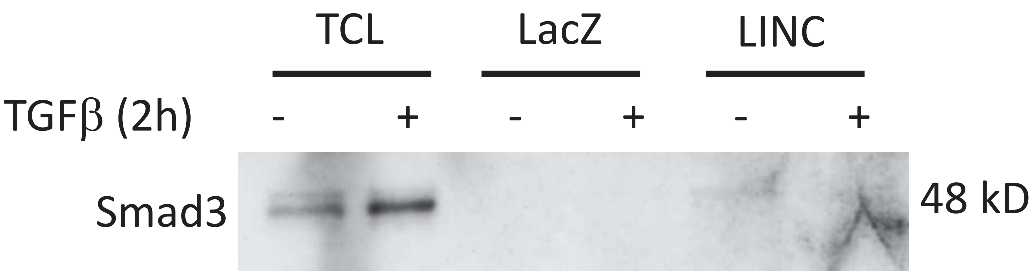

Supplemental Figure 7 related to Main Figure 7

Fig. S7 related to Fig. 7. Proteins predicted to interact with *LINC00707* and proteins predicted to regulate genes affected by *LINC00707* silencing. (**A**) Potential protein interactors of *LINC00707* obtained from the database RNAinter (http://www.rnainter.org/). The predicted interacting proteins are listed according to their score (1 for perfect match) using 0.2 as cut-off. (**B**) Potential transcription factors that bind to the promoter regions of the up-regulated genes upon stable *LINC00707* silencing in HaCaT cells (analyzed by ChEA2016). FDR<0.1. The table shows the top 20 transcription factors identified. (**C**) ChOP assay performed in PC3U cells stimulated or not with TGFβ for 2 h, using *LINC00707*-specific (LINC) or *LacZ*-specific (negative control) tiled oligonucleotides, followed by immunoblot of RNA-bound Smad3 protein. Input total cell lysate (TCL) corresponds to 10% of the lysate used for the pull-down reactions. Molecular size markers in kDa are shown. The assay is representative of four independent biological repeats.

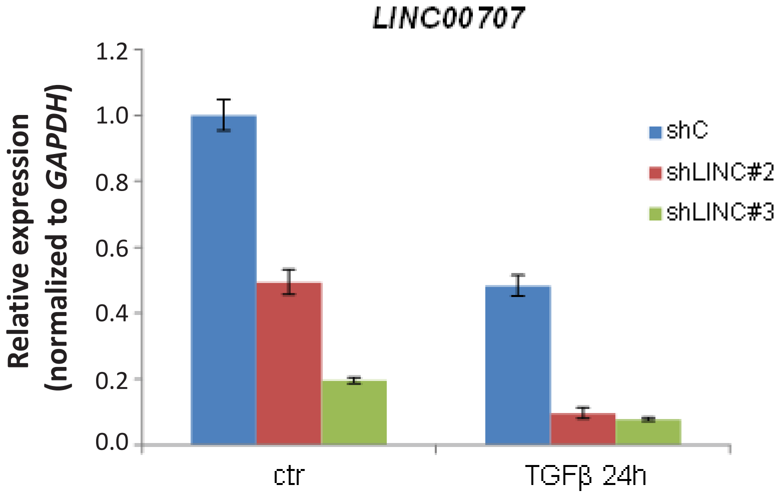

Supplemental Figure 8 related to Main Figure 9

Fig. S8 related to Fig. 9. Stable silencing of *LINC00707* in HaCaT cell clones used for transcriptomic analysis. Real-time RT-PCR for determination of *LINC00707* mRNA levels in HaCaT cell clones stably transfected with two different shRNAs targeting *LINC00707* (shLINC00707#2 or shLINC00707#3) or non-targeting shRNA (shC) and treated with TGFβ for 24 h or untreated. The graph presents data as means with SEM. No statistical analysis was performed due to the unique biological repeat, which included three technical repeats.
